# Geometrically constrained cytoskeletal reorganisation modulates DNA nanostructures uptake†

**DOI:** 10.1039/d5tb00074b

**Published:** 2025-01-16

**Authors:** Petra Elblová, Hana Andělová, Mariia Lunova, Judita Anthi, Skylar J.W. Henry, Xinyi Tu, Alexandr Dejneka, Milan Jirsa, Nicholas Stephanopoulos, Oleg Lunov

**Affiliations:** a Department of Optical and Biophysical Systems, Institute of Physics of the Czech Academy of Sciences Prague 18200 Czech Republic lunov@fzu.cz; b Faculty of Mathematics and Physics, Charles University Ke Karlovu 3 CZ-121 16 Prague 2 Czech Republic; c Institute for Clinical & Experimental Medicine (IKEM) Prague 14021 Czech Republic; d School of Molecular Sciences, Arizona State University Tempe Arizona 85287 USA nstepha1@asu.edu; e Biodesign Center for Molecular Design and Biomimetics, Arizona State University Tempe USA

## Abstract

DNA nanostructures (DNs) have gained popularity in various biomedical applications due to their unique properties, including structural programmability, ease of synthesis and functionalization, and low cytotoxicity. Effective utilization of DNs in biomedical applications requires a fundamental understanding of their interactions with living cells and the mechanics of cellular uptake. Current knowledge primarily focuses on how the physicochemical properties of DNs, such as mass, shape, size, and surface functionalization, affect uptake efficacy. However, the role of cellular mechanics and morphology in DN uptake remains largely unexplored. In this work, we show that cells subjected to geometric constraints remodel their actin cytoskeleton, resulting in differential mechanical force generation that facilitates DN uptake. The length, number, and orientation of F-actin fibers are influenced by these constraints, leading to distinct mechanophenotypes. Overall, DN uptake is governed by F-actin forces arising from filament reorganisation under geometric constraints. These results underscore the importance of actin dynamics in the cellular uptake of DNs and suggest that leveraging geometric constraints to induce specific cell morphology adaptations could enhance the uptake of therapeutically designed DNs.

## Abstract 2

DNA nanostructures (DNs) are promising for biomedical applications due to their programmability, easy synthesis, functionalization, and low toxicity. This study reveals how constrained cytoskeletal remodelling influences cellular uptake of DNs.

## Graphical abstract

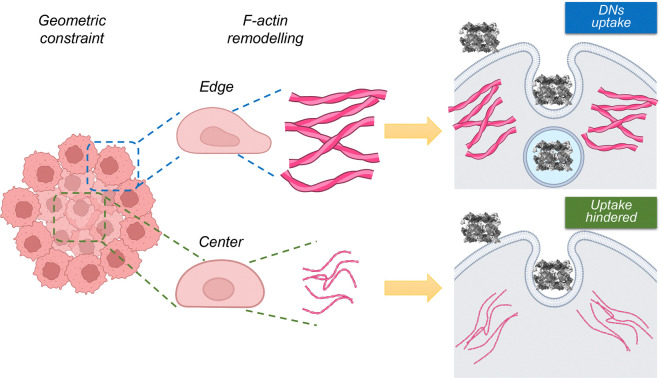

## Introduction

Cell size plays a critical role in cellular functions, affecting various physiological processes.^1–3^ Studies indicate that the rate of material uptake, including nanoparticles, is largely determined by cell size or plasma membrane surface area.^4–6^ Generally, cellular uptake has traditionally been assumed to scale with cell volume, reflecting the metabolic demands of larger cells.^4,5,7^ However, an alternative perspective suggests it correlates more closely with surface area, as uptake depends on transporter proteins and endocytic structures within the plasma membrane.^8,9^

Moreover, the geometrical organization of the cellular microenvironment shapes complex interactions between cells and regulates cellular geometry.^3,10,11^ In turn, cell geometry significantly impacts numerous cellular processes, including nuclear deformation, cytoskeleton reorganisation, chromatin compaction, gene expression, growth, apoptosis, and cell division.^12–17^ These processes are linked to the physical and biochemical cues provided by the microenvironment, emphasizing the dynamic nature of cellular behaviour within tissues.^13,16,17^ In fact, geometrically constrained cells were recently shown to exhibit different rates of nanoparticle uptake depending on the type of the constraint.^18^ A strong correlation was found between cell morphology, mechanical phenotype, and nanoparticle uptake rate, providing a basis for a novel targeting strategy for selective nanoparticle uptake.^18^ Specifically, this study demonstrated that nanoparticle uptake increases with cell spreading area, but the uptake capacity per unit area decreases with higher aspect ratios when the spreading area is constant.^18^ Additionally, cellular stress, regulated by the actin cytoskeleton, was shown to modulate membrane tension and inversely correlate with nanoparticle uptake.^18^ Actin cytoskeleton dynamics and membrane bending were identified previously as critical drivers of nanoparticle endocytosis.^19,20^ However, contrasting evidence suggests that global actin organization may not be essential for nanoparticle internalization.^21^ The findings tentatively propose that the dynamic reorganization of actin filaments likely plays a pivotal role in facilitating nanoparticle uptake.^21^

The findings from these studies suggest that nanoparticle uptake is not solely governed by biochemical signalling pathways, but is also significantly influenced by external geometric and physical cues. However, the geometric constraints in these investigations were applied at the single-cell level using micropatterning techniques, which do not account for collective cellular behaviour. Recent studies have highlighted the critical role of collective cell behaviour in essential biological processes such as cellular migration, morphogenesis, wound healing, metastasis, and embryonic development.^22,23^ Physical geometric confinement, which restricts cellular movement and spatial organization, has been shown to significantly influence cell morphology, migration, proliferation, and gene expression.^22–26^ The mechanisms by which cell crowding-induced geometric constraints regulate nanoparticle uptake, as well as the intracellular drivers of mechanical force generation required for particle engulfment, remain largely unexplored. While existing studies have predominantly focused on the role of the actin cytoskeleton and its associated mechanical forces, particularly in relation to F-actin reorganization,^18,21^ the contribution of tubulin fibres to these processes also remains underinvestigated.

DNA nanotechnology has transformed biomedicine by enabling the development of DNA nanostructures (DNs) with applications in biosensing, drug delivery, cell modulation, and bioimaging.^27–37^ While these advancements hold great potential, their successful clinical translation requires a comprehensive understanding of DN interactions with living cells and their biological consequences. Studies have extensively examined the uptake and endocytosis of DNs across various cell types,^38–44^ focusing on how physicochemical properties (*e.g.*, mass, shape, aspect ratio, size, DNA density, and surface functionalization) and cell phenotypes influence uptake.^38–46^ Although the correlation between cell size and nanoparticle uptake has been established for various nanomaterials,^5,7,18,19,47–51^ there is still a lack of studies investigating how cell size and cellular geometry may affect DN uptake.^52^ The predominant factors regulating nanoparticle uptake, such as surface area, morphology, and surface mechanics, are still a matter of debate.^18^ Moreover, information on how cell crowding-induced geometric constraints generated within a small colony of cells affect DN–cell interactions and uptake is lacking.

Interestingly, current research on the cellular uptake of DNA nanostructures (DNs) has predominantly focused on nanoparticles around ∼100 nm in size,^38–45^ with only limited investigations into smaller DNs (<20 nm).^53–55^ However, simple bundle nanostructures composed of as few as six DNA strands offer a highly promising platform for various biomedical applications.^45,52^ In particular, our recent work has demonstrated that such DNs can effectively modulate lysosomal activity.^56^ This system, based on a minimalistic six-strand bundle, presents several advantages over larger DNA origami nanostructures, which typically consist of ∼200 strands. The six-strand design is significantly more cost-effective, scalable, and potentially adaptable to *in vivo* applications, especially when the larger size and complex addressability and precise control of shape, curvature, and aspect ratio offered by DNA origami are not required. The endocytosis and cellular interactions of non-functionalized (bare) six-strand DNA bundles remain largely unexplored.

Given the limited number of studies exploring the interactions of small-sized DNs with cells, our research specifically focused on examining the behaviour of these bundles. Specifically, we aimed to determine whether and how cellular uptake of DNs is influenced by geometric constraints induced by cell crowding within small cell colonies. Furthermore, we analysed how extracellularly-driven cell geometry correlates with cytoskeletal remodelling and investigated whether actin or tubulin dynamics play a dominant role in modulating DN uptake.

Growing evidence indicates that a substantial fraction (up to 99%) of systemically administered nanoparticles are ultimately sequestered in the liver.^57,58^ Kupffer cells, the liver's resident macrophages,^57^ play a pivotal role in nanomaterial clearance. However, studies have demonstrated that nanoparticles can also directly interact with hepatocytes, highlighting the permeability of liver sinusoidal endothelial cells to nanoscale materials and the potential for direct hepatocyte engagement.^58–60^ Moreover, hepatocytes play a central role in drug and nanoparticle metabolization, and pathological conditions like hepatotoxicity and drug-induced liver injury.^61–63^ Despite this importance, the current body of literature on the interactions between DNA-functionalized nanomaterials and hepatocytes is still rather limited.^33^ In experimental research, hepatic cell lines of varying differentiation states are frequently employed to model hepatocyte functions, as primary hepatocytes derived from liver tissue are notoriously challenging to culture and maintain *ex vivo*.^62,64^ In the present study, we aimed to bridge this knowledge gap by systematically evaluating DN–cell interactions using three widely utilized hepatic cell lines: HepG2, Huh7, and Alexander cells. These models were selected based on their diverse origins and degree of hepatocyte-like functionality, providing a robust framework for elucidating the cellular mechanisms underlying DN uptake and interaction. HepG2 and Huh7 cells are particularly recognized as a robust platform for investigating the toxicological effects of various substances, including heavy metals, nanoparticles, and pharmaceuticals.^62,64,65^ Similarly, Alexander cells, also known as PLC/PRF/5 cells, are frequently employed as an alternative hepatocyte model in experimental research.^66^ While these cells also originate from liver cancer, they offer distinct phenotypic characteristics that complement the insights gained from HepG2 and Huh7 studies. Together, these cell lines provide versatile systems for modelling liver cell interactions and responses.

## Materials and methods

### Materials

Information on chemicals, fluorescent probes, antibodies, and DNA staples, including manufacturers, catalogue numbers, and dilutions, can be found in Tables S1–S4 of ESI.†

### Cell culture

In this study, we employed well-established cellular models of hepatic cells, including the human hepatoblastoma HepG2 cell line (American Type Culture Collection, ATCC) and the human hepatocellular carcinoma cell lines Alexander (PLC/PRF/5, ATCC) and Huh7 (Japanese Collection of Research Bioresources, JCRB).^67^ We utilized standard culture media, specifically EMEM medium (ATCC), supplemented with l-glutamine (BioConcept Ltd, Switzerland), 10% fetal bovine serum (Gibco™) and 1% penicillin/streptomycin (Serana Europe GmbH). Cell cultures were maintained in a humidified atmosphere with 5% CO_2_ at 37 °C, with the culture medium (EMEM) being replaced weekly. The cells were regularly screened for common contaminants, such as Mycoplasma, using the MycoAlert Detection Assay (Lonza, Switzerland). All cell lines were authenticated through short tandem repeat (STR) DNA profiling (ATCC, Manassas, VA, USA).

To subject the cells to geometric constraints, they were cultured on commercially available micropatterned surfaces featuring a covalently bound RGD motif of varying geometries, specifically μ-Slides IV 0.4 (Ibidi, Martinsried, Germany, cat. no. 80606). The cells were seeded at a concentration of 10 000 cells per 100 μL in 6-channel μ-Slides IV 0.4.

### Synthesis and characterization of DNs

DNs were synthesized as previously described.^52^ In brief, all oligonucleotides were obtained from Integrated DNA Technologies (Coralville, Iowa) and purified using 14% urea-based denaturing polyacrylamide gel electrophoresis (PAGE). Each strand was added to a mixture at a concentration of 10 μM in 1× tris-acetic acid-EDTA (TAE) buffer with 12.5 mM MgCl_2_ and annealed from 95 to 4 °C over 2 hours. The successful formation of the 6-helix bundle was confirmed using agarose gel electrophoresis. The size of the DNs was characterized using a Zetasizer Nano (Malvern Instruments). DNs were dispersed in PBS at pH 7.4. To visualize DNs by confocal microscopy, one strand of the structure was labelled with either 6-carboxyfluorescein or TAMRA fluorescent probes.

### Atomic force microscopy

Atomic force microscopy images were captured using a Bruker Multimode 8 system with a Nanoscope V controller in ScanAsyst in Fluid mode, utilizing ScanAsyst-Fluid+ AFM probes (Bruker, *k* ∼ 0.7 N m^−1^, tip radius <10 nm). A 2 μL sample was deposited on freshly cleaved mica, followed by the addition of 48 μL of 1× TAE with 12.5 mM Mg^2+^ for 2 minutes. To enhance the adsorption of DNA nanostructures on the mica surface, the surface was pre-treated using a 1 mM NiCl_2_ buffer.

### Cell viability analysis

Cell viability was assessed microscopically by monitoring the loss of plasma membrane integrity.^68^ Cells were treated with different concentrations of DNs (10, 100, and 500 nM) for 24, 48, and 72 hours. After treatment, cells were stained with propidium iodide (PI) and the nuclei were counterstained with Hoechst 33342. As a membrane-impermeable dye, PI is typically excluded from viable cells, whereas cells with compromised plasma membranes accumulate PI, staining the nuclear DNA and amplifying its fluorescence 20 to 30 times.^69^ PI staining is widely recognized as a universal indicator of cell death.^69^

Labelled cells were imaged using confocal microscopy at 200× magnification, and the numbers of dead (PI-positive) cells and total (Hoechst-stained) cells were counted using ImageJ software (NIH, Bethesda, MD, USA). Cell viability was expressed as the ratio of PI-negative cells to total cells. The viability percentage was calculated as follows:Cell viability (%) = [Number of Hoechst-stained cells − Number of PI-positive cells]/[Number of Hoechst-stained cells] × 100%

For reliable statistical sampling, we assessed 10–20 randomly selected fields per condition across three independent experiments. As a positive control, cells were treated with 20% ethanol for 60 minutes.

### High-resolution spinning disk confocal microscopy

To reveal clear subcellular details of DN localization, we utilized the IXplore SpinSR Olympus high-resolution imaging system (Olympus, Tokyo, Japan), as previously described.^52,67,70^ For cell seeding, we used 6-channel μ-Slides (Ibidi, Martinsried). Afterward, cells were treated with fluorescently labelled DNs. Specific cellular structures were then stained using fluorescent probes, as summarized in Table S2 (ESI†).

The imaging system comprises an inverted microscope (IX83; Olympus, Tokyo, Japan) and a spinning disc confocal unit (CSUW1-T2S SD; Yokogawa, Musashino, Japan). Fluorescence data for image reconstruction were collected using either a 100× silicone immersion objective (UPLSAPO100XS NA 1.35 WD 0.2 silicone lens, Olympus, Tokyo, Japan) or a 20× objective (LUCPLFLN20XPH NA 0.45 air lens, Olympus, Tokyo, Japan). The following lasers were used to excite fluorophores: 405 nm laser diode (50 mW), 488 nm laser diode (100 mW), and 561 nm laser diode (100 mW). Confocal images were acquired at a resolution of 2048 × 2048 pixels. Fluorescent images were collected using appropriate emission filters (BA420-460; BA575IF; BA510-550; Olympus, Tokyo, Japan) and captured concurrently by two digital CMOS cameras, ORCA-Flash4.0 V3 (Hamamatsu, Hamamatsu City, Japan). Fluorescence confocal images were acquired using the cellSens software (Olympus, Tokyo, Japan). Quantitative image analysis was performed by selecting approximately 5–10 random visual fields per sample, using consistent settings (*i.e.*, spinning disk speed, laser power, and offset gain). For quantitative analysis of digital images, ImageJ software (NIH, Bethesda, MD, USA) was used. An open-source software Icy (https://icy.bioimageanalysis.org)^71^ was used for 3D reconstruction and orthogonal projections visualization.

### Cellular uptake analysis by flow cytometry

Cells were treated with 6-carboxyfluorescein-labeled DNs at varying concentrations (50 and 500 nM) for different durations (24 or 48 hours). The cellular uptake of DNs was evaluated using side scatter (SSC) measurements and fluorescence assessment *via* flow cytometry. The presence of nanoparticles inside the cells increases the refractive index, which leads to an increase in side scatter light intensity.^72^ Flow cytometry measurements were conducted utilizing a CytoFLEX flow cytometer B53013 (Beckman Coulter, Brea, CA, USA). During the acquisition, 10 000 cells were collected. The acquired data were analysed using open-source software Floreada.io (https://floreada.io/analysis). Fluorescence of DNs was excited by a 488 nm laser, and data were collected at forward and side scatter, specifically in the fluorescence channel 525/40 nm (FL1). The fluorescence of 6-carboxyfluorescein was measured the FL1 channel. Cell debris, identifiable by a distinctive low forward scatter, were excluded from the analyses through gating procedures.

### Cellular uptake analysis by confocal microscopy

Additionally, we utilized confocal microscopy to assess cellular uptake of DNs. To analyze intracellular DN distribution, cells were cultured in 6-channel μ-Slides (Ibidi, Martinsried) and treated with fluorescently-labelled DNs for 24 h. For live cell imaging, the cell membrane was labelled with either CellMask™ Orange or CellMask™ Green (Thermo Fisher Scientific) plasma membrane stains. Labelled cells were visualized using spinning disk confocal microscopy IXplore SpinSR (Olympus, Tokyo, Japan), according to our verified protocols.^52,67,73^ We performed dual-color imaging for quantitative assessment of DN intracellular distribution. The stacks of confocal cross-sections obtained through confocal microscopy were analysed using the “Particle_in_Cell-3D” digital method, based on ImageJ software (NIH, Bethesda, MD, USA).^74,75^ This method automatically differentiates between intra- and extracellular spaces and was designed to analyse and quantify nanoparticle uptake by cells.^74,75^ The confocal fluorescence image of the cell membrane is converted into a mask for the cell in each measured confocal plane. By applying this mask to the corresponding particle image, nanoparticles are classified. The method distinguishes between NPs in the intracellular and extracellular spaces, as well as those near the cellular membrane, including those in transition or expanded membrane regions. Nanoparticles in these transition regions are considered to have undergone the initial step of cellular uptake. Because nanoparticles often form clusters or aggregates within the cell, counting individual particles is not feasible. Instead, fluorescence intensities are used to estimate semiquantitative the uptake of DNs.^74,75^

### Immunofluorescence labelling and remodelling assessment of cytoskeleton

Cells were subjected to geometric constraints by culturing on commercially available micropatterned surfaces featuring a covalently bound RGD motif in varying geometries, μ-Slides IV 0.4 (Ibidi, Martinsried, Germany, cat. no. 80606). Following this, the cells were fixed with a 4% paraformaldehyde solution in PBS (pH 7.4) at room temperature for 10 minutes. Cellular membranes were then permeabilized using 0.5% Triton X-100 before staining. Immunofluorescence staining was performed on fixed samples utilizing primary antibody against tubulin (Table S3, ESI†) and Alexa Fluor 568-conjugated secondary antibody (Thermo Fisher Scientific, Waltham, MA, USA). F-actin was labelled using the ActinGreen™ 488 ReadyProbes™ reagent, a high-affinity F-actin probe (phalloidin) conjugated Alexa Fluor 488 dye (Thermo Fisher Scientific). Stained cells were imaged using the spinning disk confocal microscope IXplore SpinSR (Olympus, Tokyo, Japan). Digital images were processed and quantified utilizing ImageJ software (NIH, Bethesda, MD, USA). Quantitative analysis of length and number of cytoskeleton filaments (*i.e.* F-actin and β-tubulin) was performed using the ImageJ plugin “Analyze Skeleton (2D/3D)”.^76^ Cytoskeleton filaments orientation analysis was performed using the ImageJ plugin “OrientationJ”.^77^

### Estimation of mechanical forces generated by F-actin cytoskeleton

The intracellular force generated by actin filaments (F-actin) can be modelled considering the contributions of both the length and density of these fibres.^78–81^ This relationship is complex and influenced by various factors, including the mechanical properties of the actin network, crosslinking proteins, and cellular context.^78–81^ However, a simplified conceptual equation linking intracellular force with F-actin fibre length and density can be estimated utilizing relatively simplistic model proposed previously.^80^

The dynamics of cross-linking can lead to the formation of a depletion zone near the membrane, where cross-linkers are scarce.^80,82^ This depletion zone occurs because the cross-linkers are used up or distributed unevenly, leaving a region where filaments are not effectively connected due to the insufficient availability of free cross-linkers.^80,82^ It was proposed to refer to this area as the “elastic region”.^80^ In this region, the lack of cross-linkers results in reduced filament linkage, impacting the mechanical properties and elasticity of the material.^80,82^ Indeed, it is possible to estimate the force exerted by a single filament (*f*) on the membrane using the following model:^80^*f* = *k*(*z* − *R*(*l*))where *k* is the mean spring constant of filaments, *z* is the depth of the elastic region, which we tentatively estimated as average cell length assuming force spreading through entire cell, *R* is the equilibrium length of the filament. The equilibrium length can be estimated as follows:*R* = *l*(1 − *l*/2*l*_p_)where *l* is the mean length of filaments, *l*_p_ is the persistence length of the filament. Finally, the total force *F* per whole cell can be estimated as *F* = *Nf*, with *N* being the total number of filaments per cell.^80^ The length and number of F-actin filaments were quantified using the ImageJ plugin “Analyze Skeleton (2D/3D)”.^76^ The average cell size was measured utilizing ImageJ software (NIH, Bethesda, MD, USA). The persistence length (*l*_p_) was taken as 17 μm.^81^ The spring constant of an F-actin filament varies from 0.1 to 100 pN μm^−1^.^78–81^ In order to estimate minimal force generated by single filament in this study we used the minimal value of the spring constant, or 0.1 pN μm^−1^. This model assumes a linear relationship between force, length, and density, which is a simplification. The relationship might be nonlinear and influenced by additional complex factors such as filament bundling, branching, and dynamic polymerization/depolymerization processes.^78–81,83–85^

### Statistical analysis

Cellular viability was analysed and represented as mean ± SEM. The ANOVA analysis with subsequent appropriate statistical tests was utilized to assess the statistical significance of differences between the groups. The Newman–Keuls test was used to evaluate the statistical significance of differences between multiple groups. For the two groups comparison we utilized the Mann–Whitney *U* test. Differences were considered statistically significant at (*) *P* < 0.05.

Correlation analysis between cell size and the cellular uptake of DNs, as well as between actin-generated forces and the cellular uptake of DNs, was conducted using linear regression analysis in SigmaPlot 13 software (Systat Software, Inc). Correlation coefficients and *P*-values were calculated with SigmaPlot 13 software (Systat Software, Inc). *K*-Means cluster analysis between cell size and the cellular uptake of DNs was done using OriginPro 2015 software (OriginLab Corporation). To determine the optimal number of clusters, we used the widely known “Elbow method”.^86^ This approach involves running the *K*-means algorithm for a range of *K* values and plotting the sum of squared distances from each point to its assigned cluster center (inertia) against *K*, the number of clusters.^86^ The goal is to identify the “elbow” point in the plot, where adding more clusters yields diminishing returns in reducing the sum of squared distances.^86^

Fluorescence microscopy analysis, including assessments of cell size and uptake as well as orientation, length and number of cytoskeleton filaments, was performed according to rigorously defined guidelines.^87^ Quantitative confocal microscopy assessments were carried out following established protocols and previous publications.^88–90^ Microscopy analysis involved images from three independent experiments, with each experiment including 10 randomly selected fields per sample. Sample size determination adhered to previously published statistical methods,^90,91^ which indicated that a sample size of 30 is required for a 95% confidence level and 0.9 statistical power. Therefore, at least 30 randomly selected cells were analysed for statistically robust fluorescence microscopy quantification.

Overall, the statistical methodology described in^91^ was used to determine the sample size, assuming a 95% confidence level and 0.9 statistical power.

## Results and discussion

### Characterization of DNA nanostructures

**Fig. 1:**
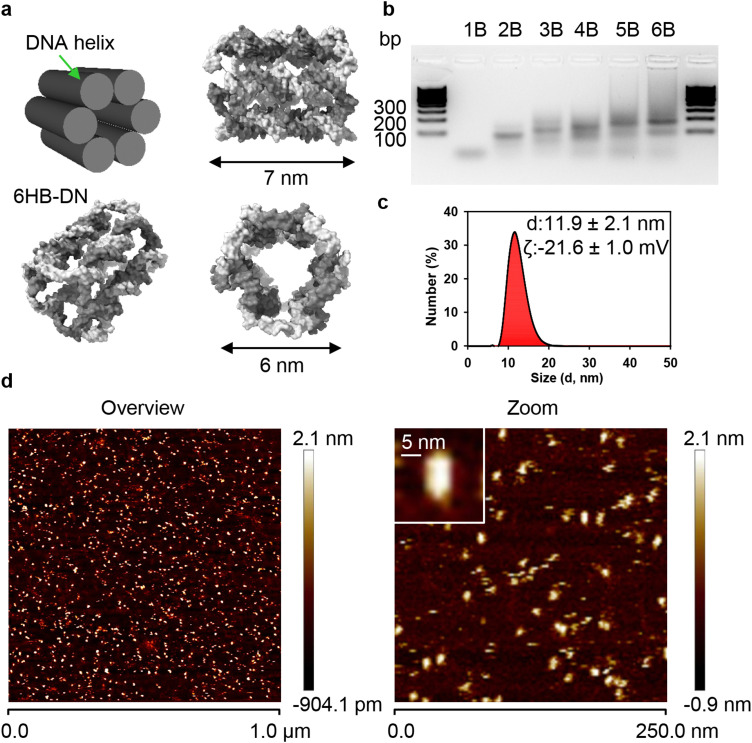
Design and characterization of 6HB-DNs. (a) Scheme of 6HB-DNs structure. (b) Agarose gel electrophoresis (1.5% agarose) used to determine the synthesis efficacy of the 6 helix bundle. (c) Size distribution and surface characterization (zeta potential) of 6HB-DNs. Characterization of the particles dissolved in PBS measured with a Zetasizer Nano (Malvern Instruments). (d) AFM characterization of the 6HB-DN.

To study the cellular uptake of DNs, we needed a robust and easily synthesized model with a relatively simple geometric shape. Our goal was to uncover fundamental aspects of cellular uptake, so we selected a DN model based on previous reports^92–94^ as a model system: a 6-helix bundle DNA nanostructure (6HB-DN) composed of six interconnected DNA duplexes (Fig. 1(a)). This type of DN forms the basis for producing multicomponent DNA bundles with programmable spatial features and compositions.^95^ Cholesterol-modified 6HB-DNs were found to be excellent structures to form nanopores in lipid membranes, thereby mediating transport of charged molecular cargos.^92–94^ In fact, the cholesterol modification made it possible to utilize 6HB as pore formation systems, minimizing endocytic uptake and maximizing incorporation in membrane lipid bilayer.^92–94^ By contrast, in our study we used non-functionalized 6HB that are actively engulfed by cells.^52,56^ In our study we used non functionalized (bare) 6HB and tested their toxicity. Importantly, the selective interaction of 6HB-DNs with different cell types was demonstrated in previous studies.^40,45,94^ Further, 6HB-DNs were shown to modulate the immune response of peripheral blood mononuclear cells (PBMCs) and granulocytes by suppressing lipopolysaccharide-induced IL-6 and TNF-α release.^45^ 6HB-DNs can also be used vehicles for co-delivering antisense oligonucleotides and silver ions into bacteria, demonstrating high antibacterial efficacy.^96^ It is worth noting that the structural stability of DNs is a crucial parameter for studying cellular interactions and uptake. Numerous studies have demonstrated that various DNA nanostructures remain structurally intact in different physiological media, and even within cells, for at least 24 hours.^45,97–101^ Specifically, we previously showed that 6HB-DNs are largely stable in PBS buffer solutions for 2 days and in lysosomal compartments for up to 24 h.^52^

Due to their structural simplicity, 6HB-DNs can be synthesized with a high production yield through a straightforward annealing process (Fig. 1(a) and (b)). The resulting bundle structure, approximately 7 × 6 nm in size, is a rigid monomeric assembly (Fig. 1(a) and (b)). Assessment of 6HB-DNs using atomic force microscopy (AFM) and dynamic light scattering (DLS) revealed that the 6HB structures in buffer solution are predominantly monodisperse, with minimal aggregation (Fig. 1(c) and (d)). Next, DLS analysis in PBS buffer solution revealed a mean hydrodynamic diameter of approximately 12 nm and a zeta potential of around −22 mV (Fig. 1(c)), indicating a stable particle solution, similar to that observed for organic nanoparticles.^102^ To quantify DN uptake by cells, one strand of the 6HB structure was labelled with either 6-carboxyfluorescein (FAM) or TAMRA fluorophores. For clarity, we refer to carboxyfluorescein-labelled 6HBs as 6HB-FAM-DNs, and TAMRA-labelled 6HBs as 6HB-TAMRA-DNs throughout the text.

### Differential uptake of 6HB-DNs by hepatic cell lines

To assess DN–cell interactions we utilized a non-trivial cell model, specifically hepatic cell lines. The liver is a primary organ that sequesters the majority of systemically injected nanomaterials.^57,58^ The liver is also an important therapeutic target for various nanoparticle-based treatments;^60,103,104^ thus, it is crucial to understand how hepatic cells interact with and engulf nanomaterials. Importantly, studies comparing and analysing DN–cell interactions in closely related cell lines remain rather limited.^33^

For an effective analysis of DN cellular uptake, it is important to first determine whether DNs exhibit cytotoxicity. In our previous work, we demonstrated that short-term exposure to 6HB-DNs at concentrations up to 500 nM does not impair the viability of Alexander, HepG2, and Huh7 hepatic cell lines.^52^ In this study we assessed potential long-term cytotoxicity of 6HB-DNs in Alexander, HepG2, and Huh7 cells. To analyse viability of three hepatic cell lines we used a propidium iodide (PI) exclusion assay. PI staining, which assesses the loss of plasma membrane integrity, is widely recognized as a universal indicator of cell death.^69^ Assessment of PI staining showed that neither short-term (*i.e.*, 24 and 48 hours) nor long-term (*i.e.*, 72 hours) treatment with 6HB-DNs at concentrations up to 500 nM exhibited any cytotoxic effects in HepG2, Huh7, and Alexander cells (Fig. S1 in ESI†).

Next, we assessed the uptake kinetics of 6HB-DNs in HepG2, Huh7, and Alexander cells. Mounting evidence suggests that the entry of nanoparticles into cells is accompanied by changes in cytoplasmic granularity, which can be analysed by flow cytometry using side scatter.^105,106^ Treatment with 6HB-DNs induced a time-dependent (Fig. S2a in ESI†) and concentration-dependant (Fig. S2b in ESI†) increase in side scatter in all three cell lines. The results clearly indicate that the side scatter distributions shift to higher values with increasing exposure times and concentrations, reflecting a higher accumulation of 6HB-DNs within the cells (Fig. S2a and b in ESI†). Of note, it is crucial to ensure that the increased cell side scattering is attributed to nanoparticles, and not cell damage or influenced by the chemical nature of the scattering nanoparticles and their state of agglomeration.^106^ Thus, to validate our results, we performed a flow cytometry assessment of the fluorescence intensity of fluorescently labelled 6HB-FAM-DNs. After incubating 6HB-FAM-DNs with all three cell lines, we evaluated the cellular uptake efficiencies and kinetics by measuring fluorescence intensity using flow cytometry. The results demonstrated similar levels of cellular internalization and kinetics as revealed by the side scatter analysis (Fig. S2c and d in ESI†). Both side scatter analysis and fluorescent intensity measurements revealed that all three cell lines saturate the uptake after 24 h of incubation (Fig. S2 in ESI†). Alexander and HepG2 cells show dose-dependent uptake that persists at high concentrations up to 500 nM, whereas Huh7 cells saturate the uptake at a 50 nM concentration (Fig. S2d in ESI†). These results provided valuable information on the timing and concentration of DN treatments to be further evaluated for mechanistic details.

**Fig. 2:**
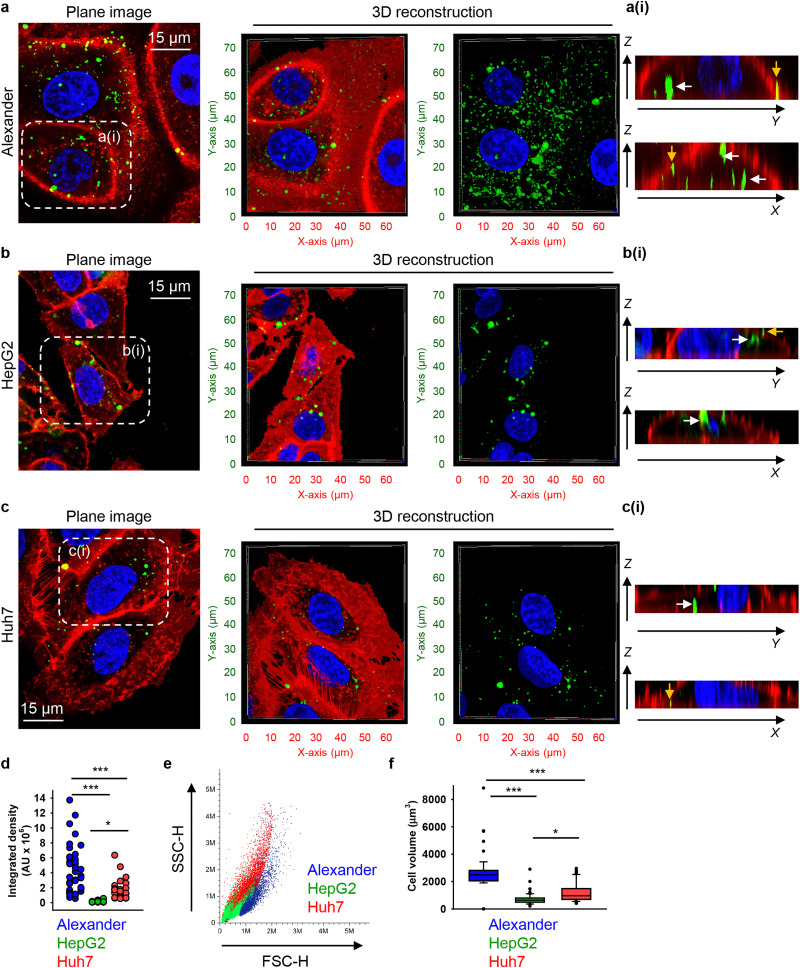
Differential uptake of 6HB-DNs by closely related cell lines. (a) Alexander, (b) HepG2, and (c) Huh7 cell lines were incubated with a 50 nM concentration of fluorescently labelled (green fluorescence) 6HB-FAM-DNs for 24 h. After the incubation, plasma membrane was labelled using CellMask Orange (Thermo Fisher Scientific) fluorescent probe. Then, cells were imaged using spinning disk confocal microscopy IXplore SpinSR (Olympus, Tokyo, Japan). 3D visualization was performed using open-source software Icy (https://icy.bioimageanalysis.org). Plane images were processed using ImageJ software (NIH). Hoechst 33342 (blue) dye was used to counterstain nuclei. Representative images from three independent experiments are presented. To visualize in details DN–cell interactions confocal *Z*-stack acquisition was processed in orthogonal projections *XZ* and *YZ*-slices of DNs interacting with cells. Rendering orthogonal projections were performed for highlighted regions of Alexander (a(i)), HepG2 (b(i)), and Huh7 (c(i)) cell lines. White arrows indicate internalized DNs, and yellow arrows show DNs attached to the membrane surface. Plane images were processed using ImageJ software (NIH). (d) Quantitative analysis of 6HB-FAM-DNs uptake by different cell lines. 6HB-FAM-DNs uptake assessment of 32–42 individual cells was performed using the ImageJ macro “Particle_in_Cell-3D”.^75^ (*) *P* < 0.05 and (***) *P* < 0.001 denote significant differences. (e) Flow cytometric analysis of cell size and granularity. (f) Quantitative analysis of cell volume of different cell lines. Cell volume assessment of 32–42 individual cells was performed using the ImageJ plugin “3D Object Counter”.^107^ (*) *P* < 0.05 and (***) *P* < 0.001 denote significant differences.

To investigate possible uptake differences between cell lines in greater detail, we performed confocal microscopy analysis with 3D reconstruction of DN accumulation in the cellular cytoplasm. High-resolution spinning disc confocal microscopy revealed that treatment for 24 h with 50 nM of 6HB-FAM-DNs resulted in a significant intracellular accumulation of the particles in all three cell types (Fig. 2(a)–(c) and Fig. S3 in ESI†). To differentiate between 6HB-FAM-DNs inside the cells and those adhered to the cell surface, cell membranes were stained (Fig. 2(a)–(c) and Fig. S3 in ESI†). To further elaborate whether DNs were internalized by the cells or merely attached to their surface, confocal *Z*-stack imaging was performed, followed by orthogonal projections in the *XZ* and *YZ* planes. These projections provided a detailed visualization of the DNs interacting with the cells (Fig. 2(a)–(c)-(ii)).

Quantitative analysis of nanoparticle uptake at the cellular level is essential for accurately evaluating the effects of nanoparticles and ensuring the efficacy of nanomedical treatments.^19,108^ In fact, microscopical 3D rendering and volumetric quantifications are crucial for correct assessment of nanoparticle uptake.^19,108,109^ Thus, we performed volumetric quantification of 6HB-FAM-DNs uptake using the ImageJ macro “Particle_in_Cell-3D”.^75^ Alexander cells exhibited a significantly higher efficiency in 6HB-FAM-DN uptake compared to Huh7 and HepG2 cells (Fig. 2(d)). HepG2 cells were the least effective in engulfing DNs (Fig. 2(d)). Mounting evidence suggests that the uptake of nanoparticles is cell-type dependent.^19,49,52,75^ However, our understanding of the comparative analysis of DN uptake in different cell lines remains rather limited.^33^ Although Alexander, HepG2, and Huh7 cells are closely related and frequently used in research as hepatocyte model surrogates, they are inherently liver cancer cells and exhibit significant genomic and transcriptomic differences.^66,110^ These differences translate into distinct sizes and morphologies of the cells (Fig. 2(e), (f) and Fig. S3 in ESI†). Alexander cells are the largest, in comparison to HepG2 and Huh7 cells (Fig. 2(e) and (f)). HepG2 cells are the smallest among the three cell lines (Fig. 2(e) and (f)), and Huh7 cells exhibit the highest granularity (Fig. 2(e)).

**Fig. 3:**
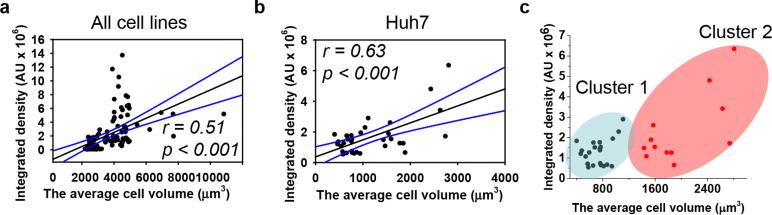
Dependence of DNs uptake on cell volume. Linear regression analysis for three cell lines (a) and Huh7 cells separately (b) between cell volume and 6HB-FAM-DN uptake. Each black point represents confocal microscopy-measured single-cell DN uptake plotted against corresponding cell volume. The uptake was measured after 24 h treatment with 50 nM concentration of 6HB-FAM-DNs. Correlation coefficients and *P* values were calculated using SigmaPlot 13 software (Systat Software, Inc). Blue lines – 95% confidence band. (c) Cluster analysis of the dependence of DNs uptake on cell volume. *K*-Means cluster analysis on the data set presented in (b) was done using OriginPro 2015 software (OriginLab Corporation).

We quantitatively assessed the cell volume of three lines, revealing that the average cell volume of Alexander cells is about 2800 μm^3^, that of HepG2 is 720 μm^3^, and the of Huh7 is 1200 μm^3^ (Fig. 2(f)). Emerging evidence suggests a possible correlation between cell size and nanoparticle uptake,^5,7,18,52^ with uptake increasing with cell size;^5,7,18,52^ however, studies addressing whether and how DN uptake in particular correlates with cell size remain limited. We have shown previously that DNs uptake positively correlates with cell area,^52^ but we did not assess DN uptake in relation to cellular volume directly. Therefore, we plotted the average cell volumes against the corresponding volumetric intracellular fluorescence of internalized 6HB-FAM-DNs (Fig. 3(a)). Regression analysis revealed a linear increase in DN uptake with cell size across all three cell lines (Fig. 3(a)). However, when we performed this analysis separately for each cell line, only Huh7 showed a statistically significant correlation between 6HB-FAM-DN uptake and cell size (Fig. 3(b) and Fig. S4a in ESI†). Further analysis of the dependency of 6HB-FAM-DNs uptake on cell volume revealed two distinct clusters of cells within the Huh7 cell line, each exhibiting different levels of uptake (Fig. 3(c) and Fig. S4b in ESI†). The Huh7 cell population is subdivided into two clusters: a cluster of larger cells showing both high and low levels of 6HB-FAM-DNs uptake, and a cluster of smaller cells exhibiting lower uptake capacities (Fig. 3(c)).

Summarizing these results, our data indicate that cell size is a poor descriptive parameter for characterizing nanoparticle uptake. Generally, there is substantial evidence supporting a positive correlation between nanoparticle uptake and cell size.^4,5,7,18,52^ However, emerging evidence suggest that while cell size may have some influence, it is overshadowed by other factors such as cell mechanics, surface properties, and intracellular pathways.^4,18,111^ For example, it was found that large cells exhibited high total cellular uptake but had a low average uptake per unit area;^4^ conversely, small cells displayed the opposite behaviour.^4^ Indeed, the larger contact area in big cells facilitates higher total cellular uptake of nanoparticles. However, the increased membrane tension in large cells demands more energy for nanoparticle engulfment, thereby reducing uptake,^4^ and these two opposing effects collectively determine the overall cellular uptake of nanoparticles.^4^ Furthermore, a recent study found that nanoparticle uptake per cell increases with a larger spreading area and decreases with a higher cell aspect ratio when the cell spreading area is constant.^18^ It is becoming evident that the mechanical properties of cells, such as stiffness and substrate adhesion, play an important role in nanoparticle uptake.^111,112^ For instance, the mechanobiology of cells significantly affects how nanoparticles interact with and are internalized by cells.^111,112^ Therefore, the current understanding of cellular nanoparticle uptake has become more complex, portraying it as a multiplex process regulated by numerous factors, including: the physicochemical characteristics of nanoparticles, protein–particle interactions and subsequent agglomeration, diffusion and sedimentation, as well as geometrically constrained mechanobiology of cells.^18,19,111,112^

### Cell crowding-induced geometric constraints affect the uptake of DNs

Considering that emerging evidence suggests cellular geometric reorganisation may modulate nanoparticle uptake,^18^ we further assessed how geometric constraints on cells might impact the DNs uptake process. Toward this end, we cultured cells on commercially available micropatterned surfaces with a covalently bound RGD motif (see Materials and methods). The geometric anisotropy of cellular constraints significantly influences the function of hepatic cells.^11^ In healthy liver tissue, hepatocytes in the sinusoidal region of the hepatic lobule are arranged linearly along sinusoidal endothelial cells, forming the hepatic cord structure.^113^ However, during cancer development, the cellular organization changes; *e.g.*, nodular hepatocellular carcinoma often exhibits a spherical or ovoid shape, characterized by well-defined margins and an expansive growth pattern.^114^ Thus, for our experiments, we selected two primary cellular organization geometries: circles and stripes.

Cells cultivated on adhesive micropatterns of various geometries conformed to the patterned shapes, forming circles with diameters of 100 μm or 200 μm and stripes with widths of 50 μm or 20 μm (Fig. S5 in ESI†). We found that 6HB-DN uptake depends on the cell's patterned shape, with cells grown on 50 μm patterns exhibiting the highest uptake (Fig. S6 in ESI†). Additionally, cells cultured on circles with a diameter of 200 μm engulfed 6HB-DNs more effectively than those on 100 μm circles (Fig. S6 in ESI†). These data clearly indicated that geometric constraints affect cellular uptake of DNs.

Fluorescent dye labelling is a common method for tracking the fate and localization of DNs within cells. However, recent findings suggest that intracellular fluorescence, including FRET signals, may not reliably indicate the uptake of intact DNA structures.^115^ Nuclease degradation both inside and outside the cell can lead to misleading fluorescence signals.^115^ In fact, DNs can be quite susceptible to intracellular enzymatic degradation.^54^ We previously demonstrated that 6HB-DNs remain stable extracellularly (in buffer conditions) for up to one month and intracellularly for up to 24 hours.^52,56^ Additionally, several studies have shown that more structurally compact DNs (*e.g.* the 6HB used here) tend to be more resistant to enzymatic degradation.^116^ However, since our primary findings were based on fluorescence quantification, we conducted control experiments to assess the uptake of TAMRA and FAM dyes alone. Both dyes exhibited very poor cell permeability, resulting in fluorescence signals comparable to those of untreated control cells (Fig. S7–S12 in ESI†). Only when high doses of ethanol were used to permeabilize the membrane did TAMRA and FAM dyes show significantly increased cellular penetration (Fig. S7–S12 in ESI†). These results support the reliability of our fluorescence-based measurements for assessing DN uptake by cells.

**Fig. 4:**
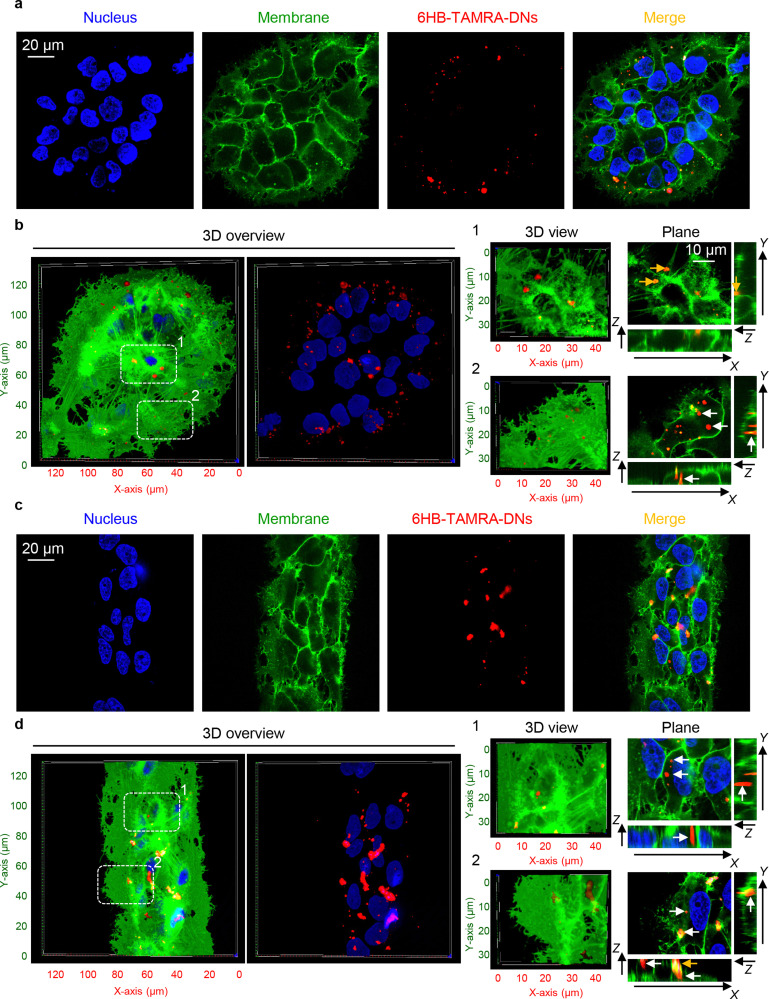
Influence of cell geometric constraint on 6HB-TAMRA-DNs uptake. Huh7 cells cultivated on adhesive micropatterns of various geometry 100 μm circles (a), (b) or 50 μm stripes (c), (d) were treated with 50 nM fluorescently labelled (red fluorescence) 6HB-TAMRA-DNs for 24 h. After the incubation, plasma membrane was labelled using CellMask Green (Thermo Fisher Scientific) fluorescent probe. Then, cells were imaged using spinning disk confocal microscopy IXplore SpinSR (Olympus, Tokyo, Japan). Hoechst 33342 (blue) dye was used to counterstain nuclei. 3D visualization and rendering orthogonal projections were performed using open-source software Icy (https://icy.bioimageanalysis.org). White arrows indicate internalized DNs, and yellow arrows show DNs attached to the membrane surface. Plane images were processed using ImageJ software (NIH).

**Fig. 5:**
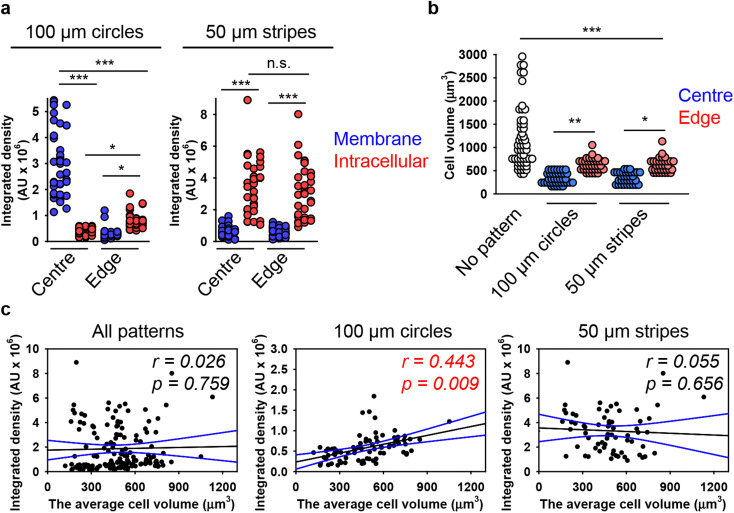
Analysis of the dependence of DNs uptake on cell volume in cells subjected to geometric constraint. (a) Quantitative analysis of 6HB-TAMRA-DNs uptake by cells subjected to geometric constraint. Huh7 cells cultivated on adhesive micropatterns of various geometry 100 μm circles or 50 μm stripes were treated with 50 nM fluorescently labelled (red fluorescence) 6HB-TAMRA-DNs for 24 h. After the incubation, plasma membrane was labelled using CellMask Green (Thermo Fisher Scientific) fluorescent probe. Then, cells were imaged using spinning disk confocal microscopy IXplore SpinSR (Olympus, Tokyo, Japan). Quantitative 6HB-TAMRA-DNs uptake assessment of 34–38 individual cells was performed using the ImageJ macro “Particle_in_Cell-3D”.^75^ (*) *P* < 0.05 and (***) *P* < 0.001 denote significant differences. (b) Quantitative analysis of cell volume of cells subjected to geometric constraint as described in (a). Cell volume assessment of 34–38 individual cells was performed using the ImageJ plugin “3D Object Counter”.^107^ (*) *P* < 0.05, (**) *P* < 0.01 and (***) *P* < 0.001 denote significant differences. (c) Linear regression analysis between cell volume and 6HB-DN uptake for Huh7 cells cultivated on adhesive micropatterns of various geometry 100 μm circles or 50 μm stripes. Each black point represents confocal microscopy-measured single-cell DN uptake plotted against corresponding cell volume. The uptake was measured after 24 h treatment with 50 nM concentration of 6HB-TAMRA-DNs. Correlation coefficients and *P* values were calculated using SigmaPlot 13 software (Systat Software, Inc). Blue lines – 95% confidence band.

Next, we hypothesized that cells within patterned shapes experience competing mechanical cues with a varying and anisotropic distribution, which affects DN uptake (Fig. S13 in ESI†). To explore the mechanistic details of this impact, we conducted experiments using cells cultured on circles with a diameter of 100 μm and stripes with a width of 50 μm. We required platforms that exhibited differences in mechanical cues at the edge compared to the centre. Circles with a diameter of 200 μm had too many cells in the centre, leading to overcrowding, while 20 μm stripes had too few cells, making it difficult to distinguish between the centre and the edge (Fig. S5 and S6 in ESI†). As suspected, we found that cells subjected to different geometrical constraint show differences in 6H-TAMRA-DNs uptake. Specifically, cells cultured at the edges of 100 μm circles exhibited different uptake compared to cells grown in the centres of the circles (Fig. 4(a) and (b)). Cells at the centres of the circles showed impaired uptake of 6HB-TAMRA-DNs, whereas cells at the edges effectively engulfed the particles. This is evident, especially from 3D reconstructions and orthogonal sections in the *x*–*z* and *y*–*z* planes, performed at a *z*-position of about half the height of the cell, which allows for clear determination of membrane-associated or intracellular DNs (Fig. 4(b), white and yellow arrows). Interestingly, cells cultured on 50 μm stripes exhibited no differences between edge and centre cells in terms of 6HB-DN uptake efficacy (Fig. 4(c) and (d)). Both edge and centre cells showed effective uptake of 6HB-DNs (Fig. 4(c) and (d)). Quantification of DN uptake confirmed the initial observations regarding the effect of different geometrical constraints on DN uptake by cells. Cells at the centres of the circles exhibited a preference for 6HB-TAMRA-DNs attached to the membrane rather than internalized (Fig. 5(a)). In contrast, edge cells showed significantly higher uptake of 6HB-TAMRA-DNs compared to centre cells, with minimal particles attached to the membranes (Fig. 5(a)). Cells cultured on 50 μm stripes exhibited no significant differences in 6HB-TAMRA-DNs uptake between central and edge cells (Fig. 5(a)). To ensure that the DNs uptake in central cells is not hindered by overgrowing of cells, we performed 3D reconstruction and orthogonal projections of cell populations cultured on patterned surfaces. This analysis confirmed that cells cultured on both 100 μm circles and 50 μm stripes grew as monolayers (Fig. S14 in ESI†).

Next, we aimed to correlate DN uptake with cell size. Quantification of cellular volume showed that cells grown on patterned surfaces had smaller volumes compared to those on non-patterned surfaces (Fig. 5(b)). Confocal images of representative cells confirmed the size differences between cells cultured on patterned and non-patterned surfaces (Fig. S15 in ESI†). Additionally, cells at the edges of both 100 μm circles and 50 μm stripes were larger than those at the centres (Fig. 5(b)). However, correlation analysis showed a very poor correlation between 6HB-TAMRA-DNs uptake and cell volume (Fig. 5(c)). These data were in line with correlation analysis of DNs uptake by cells cultured on non-patterned surfaces (Fig. 3(a)).

### Actin cytoskeleton remodelling triggered by cell crowding-induced geometric constraints drives DNs uptake by hepatic cells

It is worth noting that, on one hand, our data clearly show that geometric constraints affect DN uptake (Fig. 4 and 5(a)). On the other hand, the dependency of 6HB-TAMRA-DN uptake on cell volume does not straightforwardly explain the effect of cell geometry on particle uptake (Fig. 3 and 5). Thus, we further investigated the mechanistic explanation of how cell geometry influences particle uptake. The dynamic assembly and disassembly of cytoskeletal proteins, such as actin and tubulin, play a crucial role in modulating fundamental cellular processes like cell migration, division, and differentiation.^111,117,118^ Additionally, the cytoskeleton is a major regulator of cell morphology and mechanics.^111,119^ Cytoskeleton remodelling and membrane bending have been proposed as crucial mechanisms for cellular uptake of various nanoparticles.^20,120^ However, the specific aspects of cytoskeletal structure and dynamics relevant to this process remain unclear. Some studies suggest that actin dynamics play a more central role in actin-dependent nanoparticle endocytosis than overall actin organization.^21^ Other research indicates that nanoparticle uptake is influenced by the cellular mechanical phenotype, which is driven by the organization of cytoskeletal stress fibres and subsequent membrane stiffness.^18^ Although studies investigated the relevance of geometric constraints at the single-cell level by employing single-cell micropatterning techniques,^18–21^ this approach fails to capture the dynamics of collective cellular interactions and coordination present in multicellular systems.

**Fig. 6:**
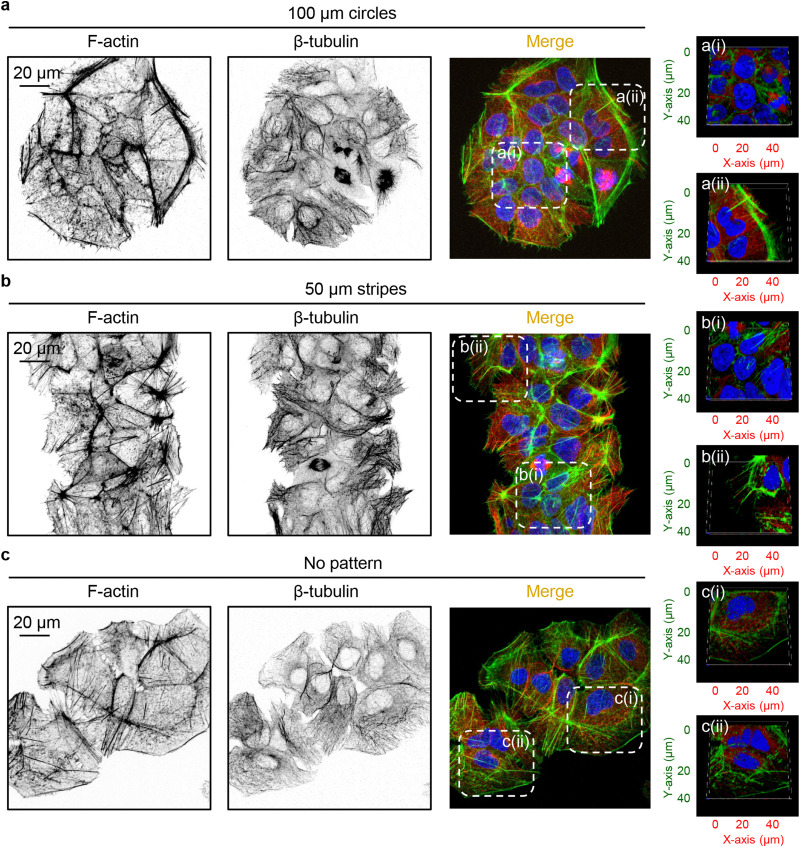
Overview of cytoskeleton organization in cells subjected to geometric constraint. Huh7 cells cultivated on adhesive micropatterns of various geometry 100 μm circles (a), 50 μm stripes (b) or non-patterned substrates (c) were stained for actin (F-actin) and tubulin (β-tubulin) filaments. 3D visualization of highlighted regions of cells cultivated on 100 μm circles (a(i) and a(ii)), 50 μm stripes (b(i) and b(ii)) or non-patterned substrates (c(i) and c(ii)) was performed using open-source software Icy (https://icy.bioimageanalysis.org).

To investigate the impact of cell crowding-induced geometrical constraints on cytoskeletal organization, we examined key components such as actin and tubulin. Staining cells with phalloidin and anti-tubulin antibodies revealed that the reorganization of actin and tubulin fibres was closely associated with alterations in cell shape when cultured on patterned surfaces (Fig. 6(a) and (b)). Notably, the geometrical constraints imposed by cell crowding led to significant cytoskeletal remodelling of both actin and tubulin compared to cells cultured on non-patterned surfaces (Fig. 6).

**Fig. 7:**
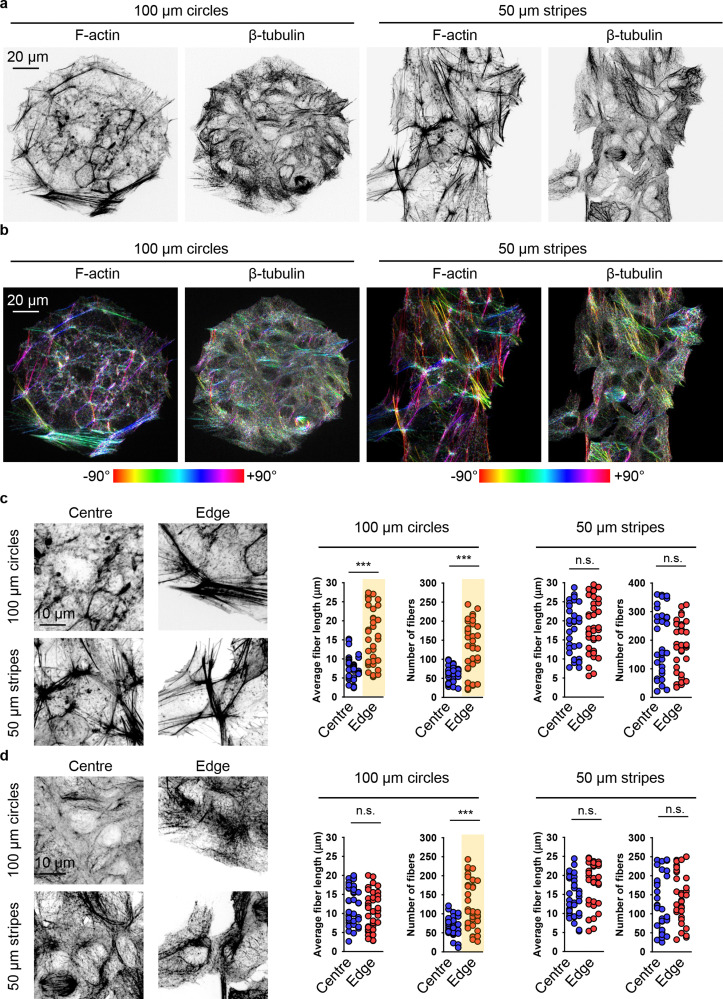
Cytoskeleton re-organization analysis in cells subjected to geometric constraint. (a) Huh7 cells cultivated on adhesive micropatterns of various geometry 100 μm circles or 50 μm stripes were stained for actin (F-actin) and tubulin (β-tubulin) filaments. (b) Corresponding orientation plots for actin and tubulin staining, where the different colours indicate different orientations of cytoskeleton filaments as per the given colourmap. Orientation analysis was performed using the ImageJ plugin “OrientationJ”.^77^ Assessment of length and number of actin (c) and tubulin (b) filaments in cells subjected to geometric constraint as described in (a). Quantitative analysis of length and number of cytoskeleton filaments of 34–38 individual cells was performed using the ImageJ plugin “Analyze Skeleton (2D/3D)”.^76^ (***) *P* < 0.001 denote significant differences.

Further, we investigated in detail how cell crowding-induced geometrical constraints affect cytoskeletal dynamics and intracellular distribution. Cells at the centre of 100 μm circles did not form significant amounts of F-actin stress fibres and predominantly exhibited a non-polarized, circular shape with an orthoradial pattern of actin filaments (Fig. 7(a)). In contrast, cells at the edges of the 100 μm circles displayed prominent F-actin stress fibres (Fig. 7(a)). A similar reorganisation was observed in the tubulin cytoskeletal elements (Fig. 7(a)). On 50 μm stripes, cells displayed well-developed F-actin stress fibres, which were preferentially aligned in the same direction (Fig. 7(a)). Cells at both the edge and centre exhibited a similar degree of F-actin fibre re-organization and alignment (Fig. 7(a)). Tubulin cytoskeletal remodelling followed F-actin in cells cultured on 50 μm stripes (Fig. 7(a)). Next, we assessed the local angles of cytoskeletal filaments, as shown in the coloured orientation plots (Fig. 7(b)). Cells at the edges of 100 μm circles exhibited uniformly oriented F-actin fibres compared to those at the centre (Fig. 7(b)). Interestingly, tubulin fibres did not exhibit the same trend (Fig. 7(b)). In cells cultured on 50 μm stripes, both F-actin and tubulin fibres demonstrated a high degree of ordering in both edge and centre cells (Fig. 7(b)).

Detailed quantitative analysis of cytoskeletal remodelling revealed that F-actin fibres were both longer and more numerous in edge cells compared to central cells cultured on 100 μm circles (Fig. 7(c)). In contrast, cells cultured on 50 μm stripes exhibited no significant differences in the number or size of either F-actin stress fibres or tubulin fibres between edge and central cells (Fig. 7(c) and (d)). Notably, tubulin fibres in edge cells compared to central cells on 100 μm circles showed no difference in fibre length, although these fibres were found to be more numerous in edge cells (Fig. 7(d)).

**Fig. 8:**
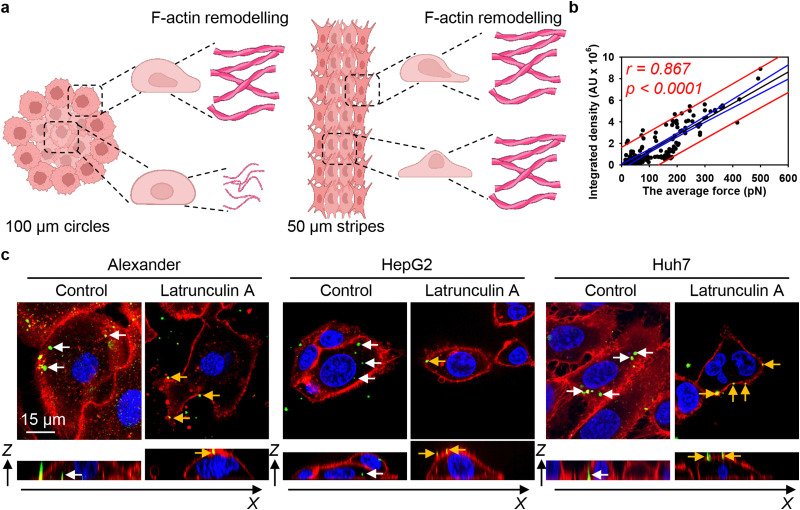
F-actin cytoskeleton re-organization drives 6HB-DNs uptake in cells subjected to geometric constraint. (a) Schematics of F-actin remodelling in cells subjected to geometric constraint. Created with https://BioRender.com. (b) Linear regression analysis average mechanical force generated by F-actin filaments and 6HB-DN uptake. Each black point represents confocal microscopy-measured single-cell DN uptake plotted against corresponding estimated mechanical force generated by F-actin filaments. The uptake was measured after 24 h treatment with 50 nM concentration of 6HB-TAMRA-DNs. Correlation coefficients and *P* values were calculated using SigmaPlot 13 software (Systat Software, Inc). Blue lines – 95% confidence band, red lines – 95% prediction band. (c) DNs uptake inhibition by latrunculin A. Alexander, HepG2, and Huh7 cell lines were incubated with a 50 nM concentration of fluorescently labelled (green fluorescence) 6HB-FAM-DNs in the presence or absence of 100 nM latrunculin A for 24 h. After the incubation, plasma membrane was labelled using CellMask Orange (Thermo Fisher Scientific) fluorescent probe. Then, cells were imaged using spinning disk confocal microscopy IXplore SpinSR (Olympus, Tokyo, Japan). Rendering orthogonal projections were performed using open-source software Icy (https://icy.bioimageanalysis.org). White arrows indicate internalized DNs, and yellow arrows show DNs attached to the membrane surface. Plane images were processed using ImageJ software (NIH).

These data can be interpreted in light of mounting evidence that actin filaments are the primary force-generating machinery in the cell, producing pulling forces that drag nanoparticles into the cytosol.^20,120,121^ We hypothesize that the differential reorganisation of F-actin filaments at the edges and centres of patterned surfaces would lead to variations in mechanical forces, thereby influencing DN uptake by the cells (Fig. 8(a)). Thus, we estimated the pulling force of actin filaments using a previously published model that accounts for both the length and density of these fibers.^80^ We then correlated the actin-generated forces with 6HB-DN uptake. Regression analysis revealed a linear increase in DN uptake with increasing actin-generated force (Fig. 8(b)). Importantly, the range of forces, depending on the number and length of filaments, was estimated to be between approximately 10 and 500 pN (Fig. 8(b)), a range consistent with the measured tension (pulling) forces within the cell.^122^ In fact, the correlation between 6HB-DN cellular uptake and actin-generated forces was statistically significant and showed a better agreement with the data compared to the correlation with cellular volume (Fig. 3(b), 8(b) and Fig. S4a in ESI†). To validate these findings and confirm the involvement of F-actin in DN uptake, we analysed 6HB-DN uptake in the presence of a potent F-actin polymerization inhibitor, latrunculin A. Administration of latrunculin A significantly reduced 6HB-FAM-DN uptake across three cell lines, resulting in predominantly membrane-bound DNs (Fig. 8(c) and Fig. S16 in ESI†), confirming the crucial role of F-actin-generated forces in DN uptake.

It is important to highlight that we approached the problem of DN cellular uptake from a cell mechanics perspective. While this is a key factor, the uptake process is far more complex and involves various other players and signalling pathways. Interestingly, a mechanistic approach to cellular uptake offers certain advantages. For instance, the F-actin cytoskeleton has been implicated in all kinetically distinguishable forms of endocytosis.^19,123^ Therefore, F-actin remodelling can serve as a predictable factor for assessing DN uptake. Our study highlights this point, demonstrating that constraint-driven F-actin remodelling regulates DN uptake even within the same cell line population.

## Conclusions

Although the past two decades showed marked progress in understanding of the fundamental processes that mediate nanoparticle–cell interactions, further studies are needed to reveal the mechanics of nanoparticle interactions with biological environments.^124,125^ In the context of DNA-based nanotechnology, the impact of cell morphology and geometric constraints on DN–cell interactions, particularly uptake, remains underrepresented in the current literature.

In this study, we found that the uptake of DNs is proportional to the volume of the cell. However, cells constrained by micropatterned surfaces exhibited a weak dependence of DNs uptake on cell volume. In fact, the uptake of DNs by geometrically constrained cells was linearly dependant on the force generated by the remodelled actin cytoskeleton. Specifically, cells bearing longer, and thicker actin filaments were found to engulf DNs more effectively. Pharmacological inhibition of actin polymerization in cells abrogated DN uptake, confirming the pivotal role of the actin cytoskeleton in this process. Contrary to single-cell micropatterning techniques,^18–21^ we investigated how cell crowding-induced geometric constraints within a small colony of cells affect DN–cell interactions. We revealed that such that changes in cell geometry driven by such constraints is associated with internal actin cytoskeleton remodelling, which in turn causes change in 6HB-DN uptake. We found that cells subjected to geometric constraints remodel their actin cytoskeleton, resulting in differential mechanical force generation that drags 6HB-DNs into the cell. The length, number, and orientation of F-actin fibres are governed by the geometric constraints produced by micropatterned surfaces, leading to the formation of distinct mechanophenotypes of cells. We found that anisotropy of cell crowding-induced geometric constraints modulates actin cytoskeleton remodelling and consequently DN uptake. The circular geometry of cell crowding-induced geometric constraints resulted in distinct reorganization of F-actin in cells grown in the centre and edge of the patterns, which resulted in differences in DN uptake by central and edged cells. Thus, our work demonstrates that constraint-driven F-actin remodelling regulates DN uptake, even within the same cell line population.

Indeed, it was recently found that F-actin architecture determines the effectiveness of mechanical work performance.^84^ In line with these findings, we show that DN uptake is governed by F-actin forces originating from filament reorganisation under geometric constraints. Additionally, it was recently found that distinct cell morphologies, exhibiting different F-actin filament organizations, can be targeted with nanoparticles, enabling a novel type of targeting called mechanotargeting.^18^ Here we tentatively show the feasibility of such targeting utilizing DNs. We hope that presented here results could serve as a foundation for the rational design of DNs for various biomedical applications, including potential use in effective targeting for mechanobiologically relevant diseases.

## Author contributions

Oleg Lunov and Nicholas Stephanopoulos conceived the idea and designed the study. Oleg Lunov performed conceptualization, methodology, data curation, funding acquisition, supervision, and writing – original draft. Petra Elblová carried out most of the experiments and analysed the data. Mariia Lunova and Milan Jirsa contributed to methodology, data analysis, provided scientific suggestions. Hana Andělová and Petra Elblová performed immunostaining and confocal imaging analysis. Petra Elblová and Judita Anthi performed 6HB synthesis and gel electrophoresis. Xinyi Tu and Skylar J. W. Henry conducted the synthesis of nanomaterials and corresponding characterizations. Mariia Lunova, Milan Jirsa, Nicholas Stephanopoulos and Alexandr Dejneka discussed and revised the manuscript. All authors contributed to the discussion of the results and agreed with the results. All the authors contributed to writing the manuscript.

## Data availability

The datasets generated and analyzed during the current study are available from the corresponding author on reasonable request. All relevant data supporting the findings of this study, including experimental results, imaging data, and statistical analyses, can be provided upon request to ensure transparency and reproducibility.

## Conflicts of interest

There are no conflicts to declare.

## Supplementary Material

TB-013-D5TB00074B-s001
