# Supplementary material for: Geometrically constrained cytoskeletal reorganisation modulates DNA nanostructures uptake
Source: J Mater Chem B. 2025 Jan 16;13(7):2335–51. doi: 10.1039/d5tb00074b (PMC11749194; doi:10.1039/d5tb00074b)
Supplement: TB-013-D5TB00074B-s001 [file TB-013-D5TB00074B-s001.pdf]

Supplementary Information (SI) for Journal of Materials Chemistry B.
This journal is © The Royal Society of Chemistry 2025

Supplementary Material

Geometrically constrained cytoskeletal reorganisation modulates DNA nanostructures uptake

Petra Elblová[a,b], Hana Andělová[a], Mariia Lunova[a,c], Judita Anthi[a], Skylar J.W. Henry[d,e], Xinyi Tu[d,e], Alexandr Dejneka[a], Milan Jirsa[c], Nicholas Stephanopoulos[,d,e] and Oleg Lunov*[,a]*

[a] Department of Optical and Biophysical Systems, Institute of Physics of the Czech Academy of Sciences, Prague, 18200, Czech Republic.

[b] Faculty of Mathematics and Physics, Charles University, Ke Karlovu 3, CZ-121 16 Prague 2, Czech Republic.

[c] Institute for Clinical & Experimental Medicine (IKEM), Prague, 14021, Czech Republic.

[d] School of Molecular Sciences, Arizona State University, Tempe, Arizona 85287, United States.

[e] Biodesign Center for Molecular Design and Biomimetics, Arizona State University, Tempe, United States.

* Email: nstepha1@asu.edu; * Email: lunov@fzu.cz

Table S1. List of chemicals used in the study.

Reagent	Catalog number	Manufacturer
IDTE pH 7.5 (1X TE Solution)	11-05-01-05	Integrated DNA Technologies
GeneRuler™ 100bp DNA Ladder Plus	SM0321	Thermo Fisher Scientific
Agarose SERVA for DNA electrophoresis	11404	Serva
SYBR™ Gold Nucleic Acid Gel Stain (10 000X Concentrate in DMSO)	S11494	Thermo Fisher Scientific
Purple Loading Dye, Purple (6X), no SDS	B7025S	New England Biolabs
UltraPure™ Ethidium Bromide, 10 mg/mL	15585011	Thermo Fisher Scientific
Tris Buffered Saline	T5030-100TAB	Sigma Aldrich
EDTA (Komplexon II)	E9884	Penta
Magnesium chloride hexahydrate	16330-31000	Penta
Acetic acid solution 1M (1N) Titripur®	1.60305	Supelco
Latrunculin A	428021	Sigma Aldrich
L-glutamine	1-31S01-I	BioConcept Ltd.
fetal bovine serum	10500-064	Gibco
Penicilin-streptomycin	RAL-001-100ML	Serana Europe GmbH
Trypsin-EDTA	LM-T1706/500	Biosera
PBS	10010-015	Gibco
5-Carboxytetramethylrhodamine (TAMRA)	C2734	Sigma Aldrich
Fluorescein isothiocyanate isomer I (FAM)	F7250	Sigma Aldrich

Table S2. List of fluorescent probes used in the study.

Reagent	Catalog number	Dilution	Manufacturer
Hoechst 33342 Solution (20 mM)	62249	0,005 mg/mL	Thermo Fisher Scientific
Propidium Iodide Ready Probes™ Reagent	R37108	2 drops/mL	Thermo Fisher Scientific
CellMask™ Orange Plasma Membrane Stain	C10045	1:1000	Thermo Fisher Scientific
CellMask™ Green Plasma Membrane Stain	C37608	1:1000	Thermo Fisher Scientific

Table S3. List of antibodies used in the study.

Antibody	Clone/catalogue No	Dilution	Manufacturer
Anti- β-Tubulin	D2N5G/ 15115	1:100	Cell Signaling Technology
AlexaFluor 568 goat anti-rabbit IgG	A-11011	1:1000	Thermo Fisher Scientific

Table S4. List of DNA staples used in the study.

DNA staples	Sequence 5‘ -> 3‘	Manufacturer
**6HB 1**	AGC GAA CGT GGA TTT TGT CCG ACA TCG GCA AGC TCC CTT TTT CGA CTA TT	Integrated DNA Technologies
**6HB 2**	CCG ATG TCG GAC TTT TAC ACG ATC TTC GCC TGC TGG GTT TTG GGA GCT TG	
**6HB 3**	CGA AGA TCG TGT TTT TCC ACA GTT GAT TGC CCT TCA CTT TTC CCA GCA GG	
**6HB 4**	AAT CAA CTG TGG TTT TTC TCA CTG GTG ATT AGA ATG CTT TTG TGA AGG GC	
**6HB 5**	TCA CCA GTG AGA TTT TTG TCG TAC CAG GTG CAT GGA TTT TTG CAT TCT AA	
**6HB 6**	CCT GGT ACG ACA TTT TTC CAC GTT CGC TAA	

	TAG TCG ATT TTA TCC ATG CA	

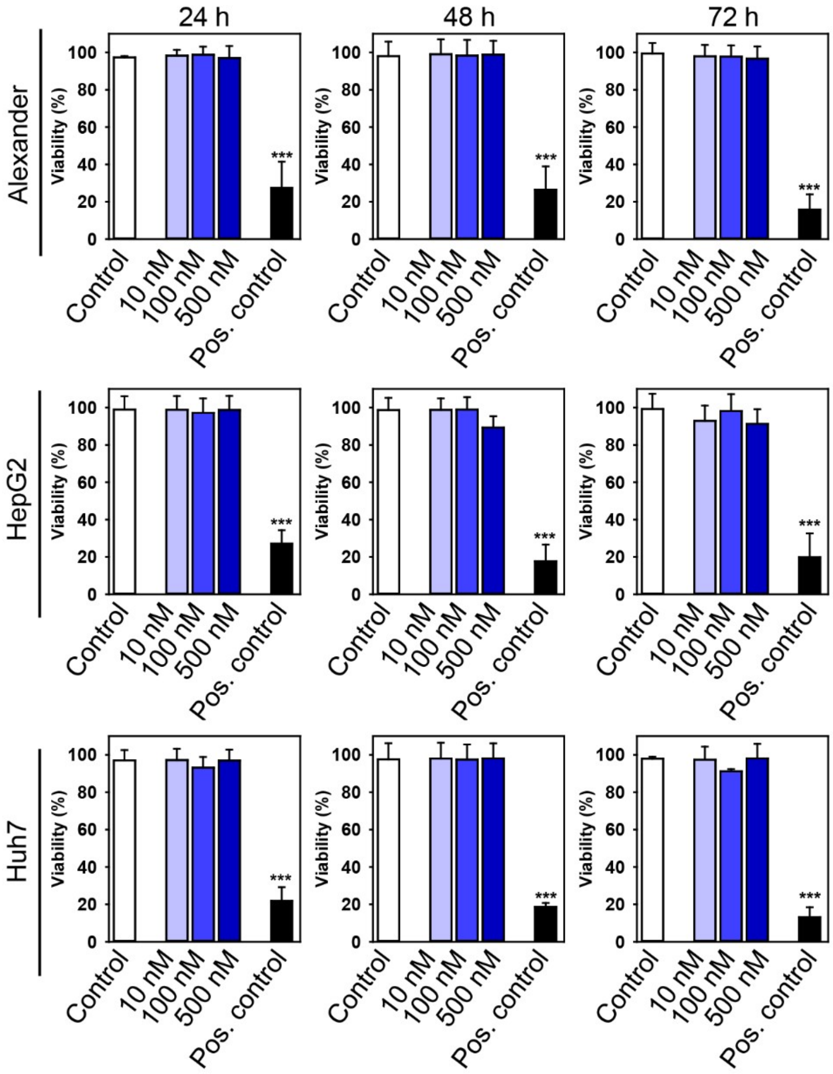

Figure S1. 6HB-DNs do not induce toxicity in hepatic cell lines. The viability of Alexander, HepG2 and Huh7 cells was checked using propidium iodide exclusion assay. Cells were treated with 6HB-DNs (10, 100 and 500 nM) for 24, 48 and 72 h. After the treatment, cells were stained with propidium iodide (PI) and nuclei were counterstained with Hoechst 33342. Labelled cells were then imaged by spinning disk confocal microscopy IXplore Spin SR (Olympus, Tokyo, Japan), and the numbers of dead (PI-positive) cells and total number (Hoechst-stained) of cells were counted using the ImageJ software (NIH). The viability was expressed as the ratio of PI-negative cells to total cells. As a positive control, cells were treated with 20% ethanol for 20 min.

Data are expressed as means ± SEM (n = 3). (***) $P < 0.001$ denotes statistically significant differences.

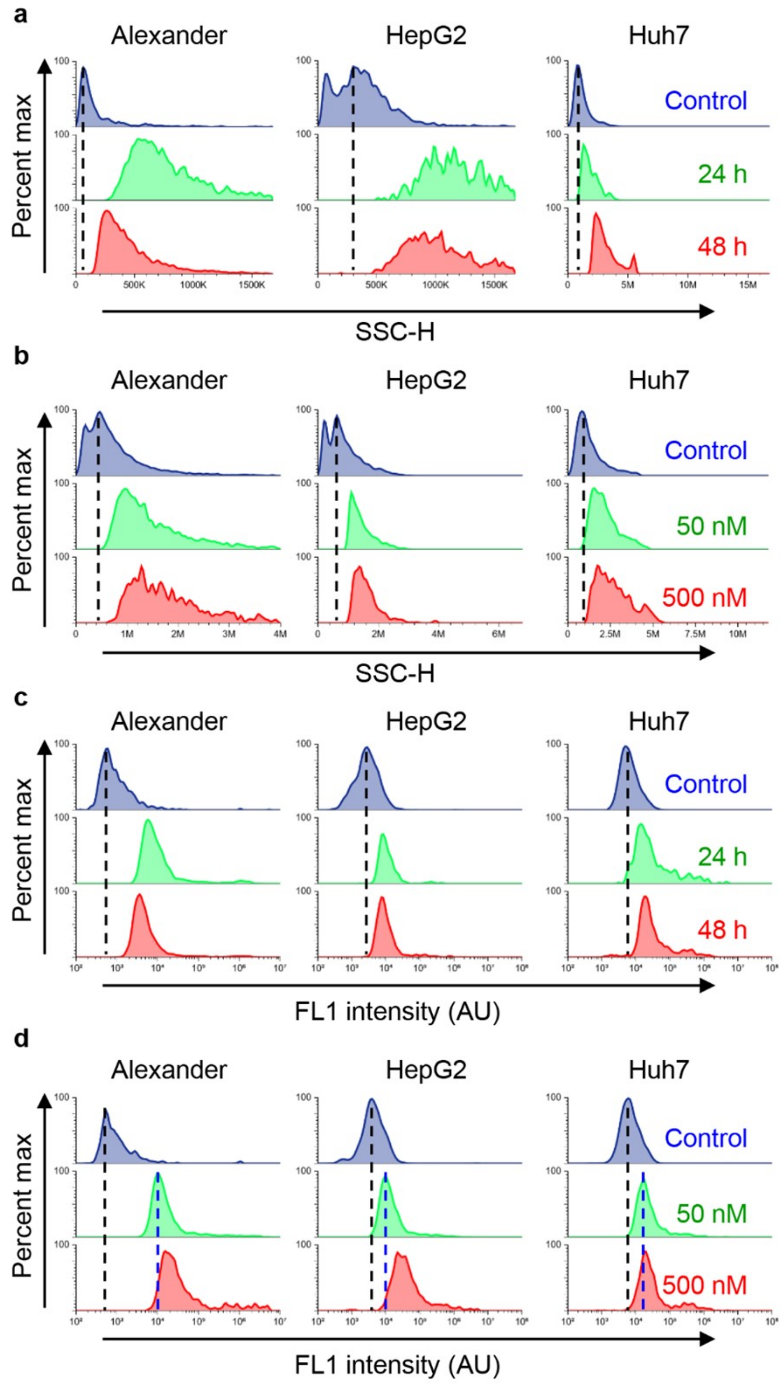

Figure S2. Cellular DNs uptake studies by flow cytometry. (a,b) Measuring 6HB-DNs uptake by changes in cell side scattering. Alexander, HepG2 and Huh7 cells were treated with 50 nM of

6HB-DNs for 24 or 48 h (a). Alternatively, cells were treated with different concentrations of 6HB-DNs (50 and 500 nM) for 48 h (b). Side-scattering was measured by flow cytometry. Control refers to untreated cells not exposed to nanoparticles. (c,d) Alexander, HepG2 and Huh7 cells were exposed to different concentration of 6HB-FAM-DNs (50 and 500 nM) for the indicated times prior to measurement of the fluorescence intensity of 6HB-FAM-DNs by flow cytometry. Control refers to untreated cells not exposed to nanoparticles.

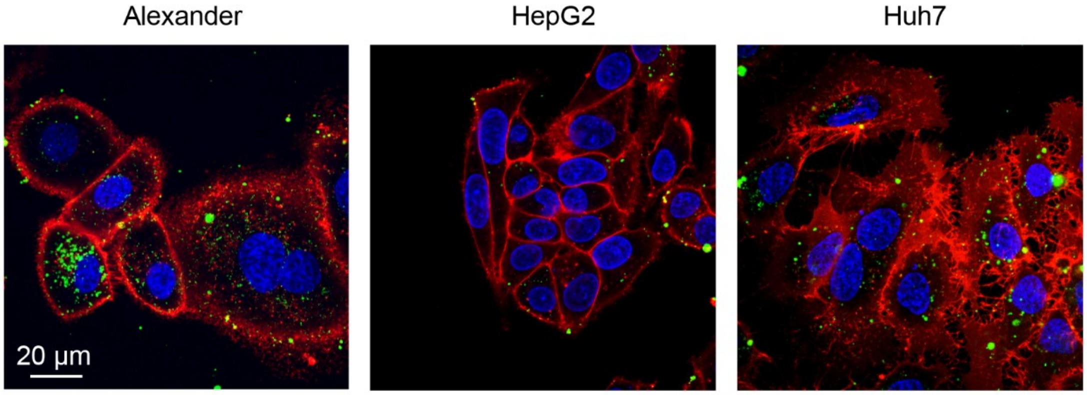

Figure S3. Differential uptake of 6HB-DNs by closely related cell lines. Alexander, HepG2, and Huh7 cell lines were incubated with a 50 nM concentration of fluorescently labelled (green fluorescence) 6HB-FAM-DNs for 24 h. After the incubation, plasma membrane was labelled using CellMask Orange (Thermo Fisher Scientific) fluorescent probe. Then, cells were imaged using spinning disk confocal microscopy IXplore SpinSR (Olympus, Tokyo, Japan). Hoechst 33342 (blue) dye was used to counterstain nuclei. Plane images were processed using ImageJ software (NIH). Representative images from three independent experiments are presented.

a

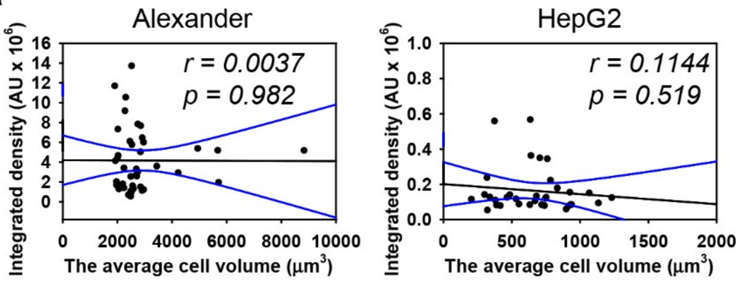

b

	Cell volume (μm^3)	Integrated density (AU x 10^6)
Cluster 1	723	1.29
Cluster 2	1978	2.34

Figure S4. Dependence of DNs uptake on cell volume. (a) Linear regression analysis for Alexander and HepG2 cells separately between cell volume and 6HB-FAM-DN uptake. Each black point represents confocal microscopy-measured single-cell DN uptake plotted against corresponding cell volume. The uptake was measured after 24 h treatment with 50 nM concentration of 6HB-FAM-DNs. Correlation coefficients and P values were calculated using SigmaPlot 13 software (Systat Software, Inc). Blue lines – 95% confidence band. (b) Cluster analysis of the dependence of DNs uptake on cell volume. K-means cluster analysis on the data set presented in (Figure 3b) was done using OriginPro 2015 software (OriginLab Corporation).

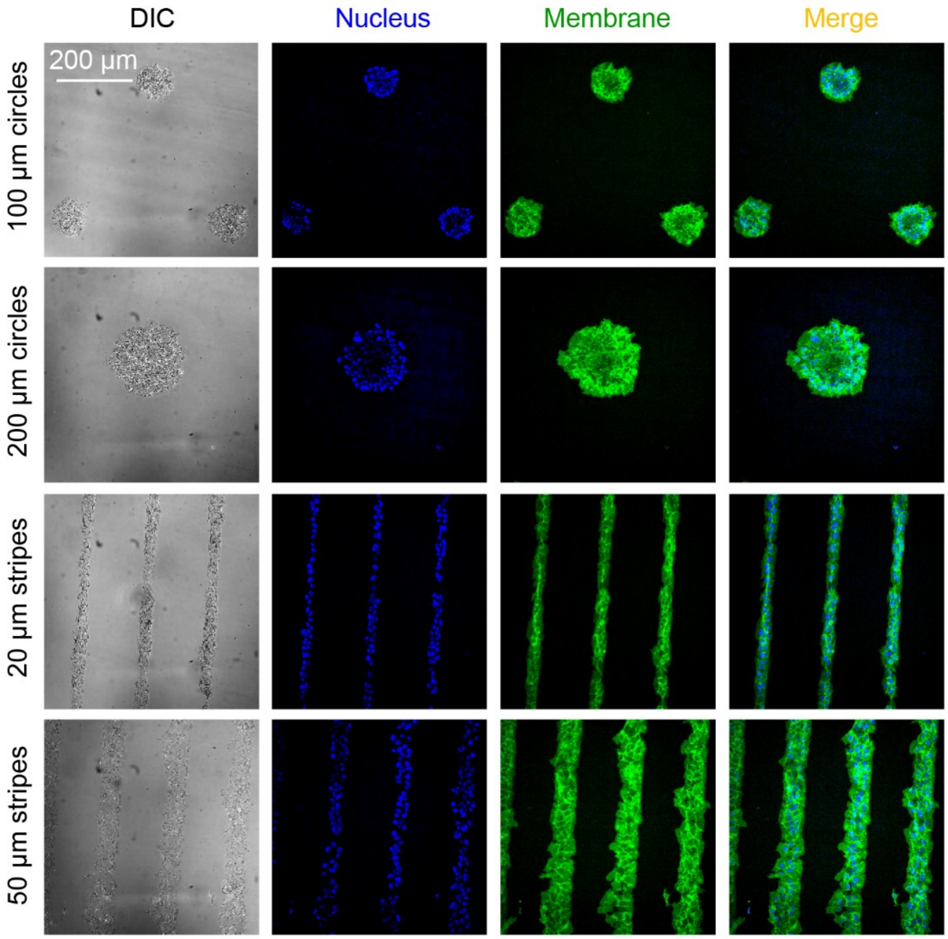

Figure S5. Cell growth under geometric constraints. Huh7 cells were cultivated on adhesive micropatterns of various geometry (μ-Slide VI 0,4 μ-Pattern^{RGD} Test Patterns 1 (ibidi, Martinsried, Germany, cat. no. 83651), e.g. circles (diameter: 100 μm or 200 μm) or stripes (width: 50 μm or 20 μm). Plasma membrane was labelled using CellMask Green (Thermo Fisher Scientific) fluorescent probe. Then, cells were imaged using spinning disk confocal microscopy IXplore SpinSR (Olympus, Tokyo, Japan). Hoechst 33342 (blue) dye was used to counterstain nuclei.

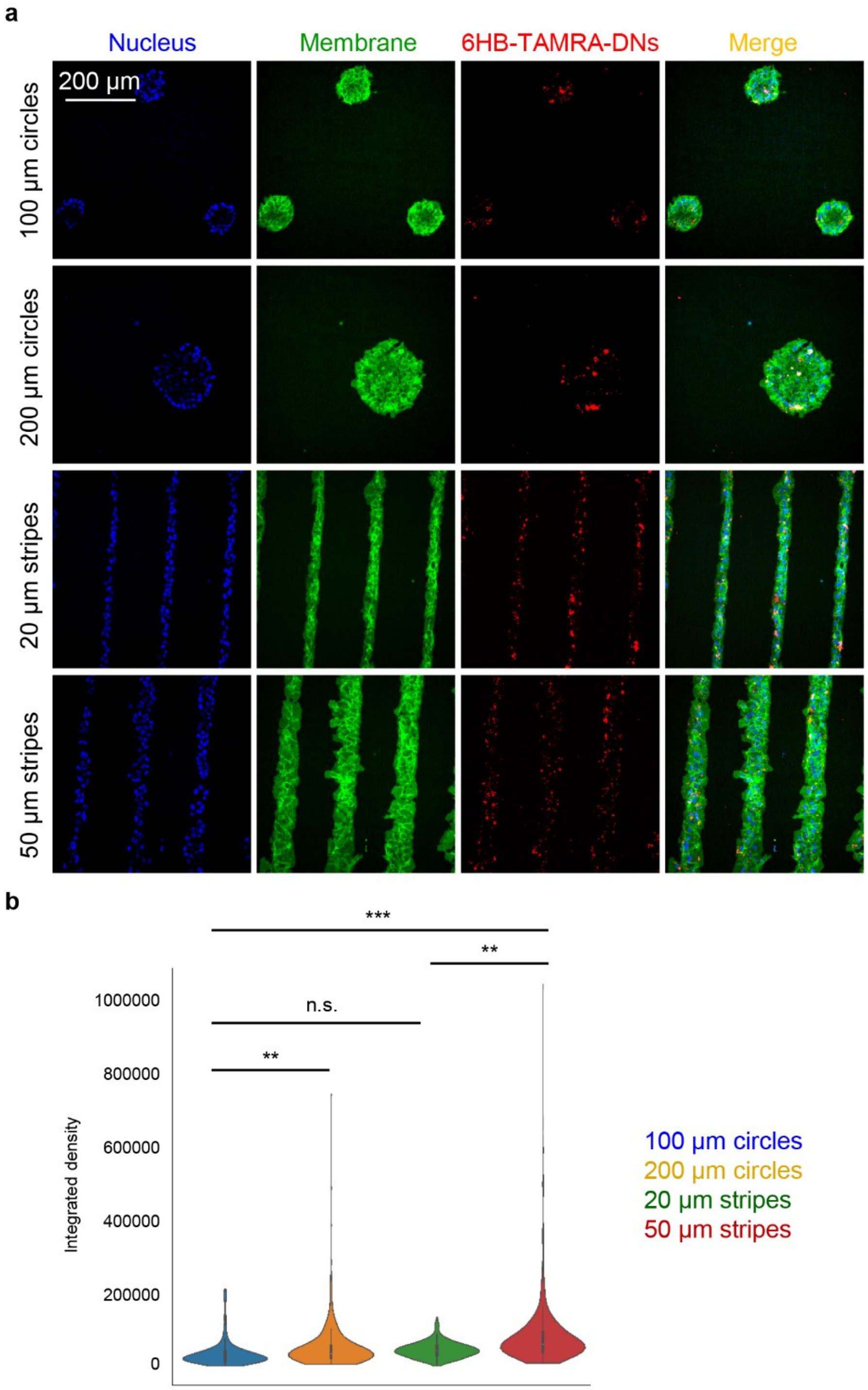

Figure S6. Influence of cell geometric constraint on 6HB-TAMRA-DNs uptake. (a) Huh7 cells were cultivated on adhesive micropatterns of various geometry (µ-Slide VI 0,4 µ-Pattern^{RGD} Test Patterns 1 (ibidi, Martinsried, Germany, cat. no. 83651), e.g. circles (diameter: 100 µm or 200 µm)

or stripes (width: 50 μm or 20 μm). After, cells were treated with 50 nM fluorescently labelled (red fluorescence) 6HB-TAMRA-DNs for 24 h. Following the incubation, plasma membrane was labelled using CellMask Green (Thermo Fisher Scientific) fluorescent probe. Then, cells were imaged using spinning disk confocal microscopy IXplore SpinSR (Olympus, Tokyo, Japan). Hoechst 33342 (blue) dye was used to counterstain nuclei. (b) Quantification of 6HB-DNs uptake in cells cultivated on adhesive micropatterns of various geometry, as described in (a). Plane images were processed and quantified using ImageJ software (NIH). N = 209-567 individual cells were assessed. Violin plots were created using open-source software (http://www.bioinformatics.com.cn/login_en/).

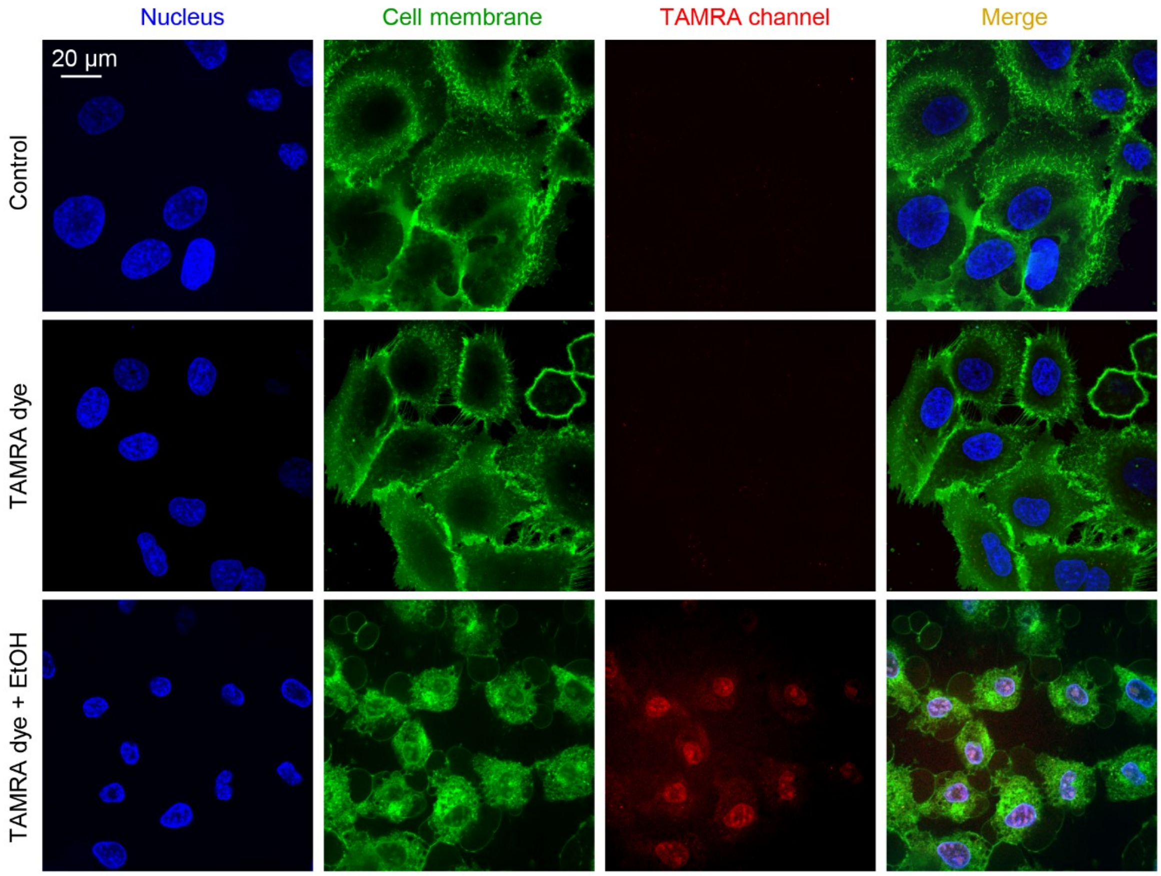

Figure S7. TAMRA dye uptake by Alexander cells. Cells were incubated with a 50 nM concentration of TAMRA dye for 24 h. After the incubation, plasma membrane was labelled using

CellMask Green (Thermo Fisher Scientific) fluorescent probe. Then, cells were imaged using spinning disk confocal microscopy IXplore SpinSR (Olympus, Tokyo, Japan). Hoechst 33342 (blue) dye was used to counterstain nuclei. Plane images were processed using ImageJ software (NIH). To highlight membrane impermeability of TAMRA, cells were stressed with 20% of ethanol (EtOH).

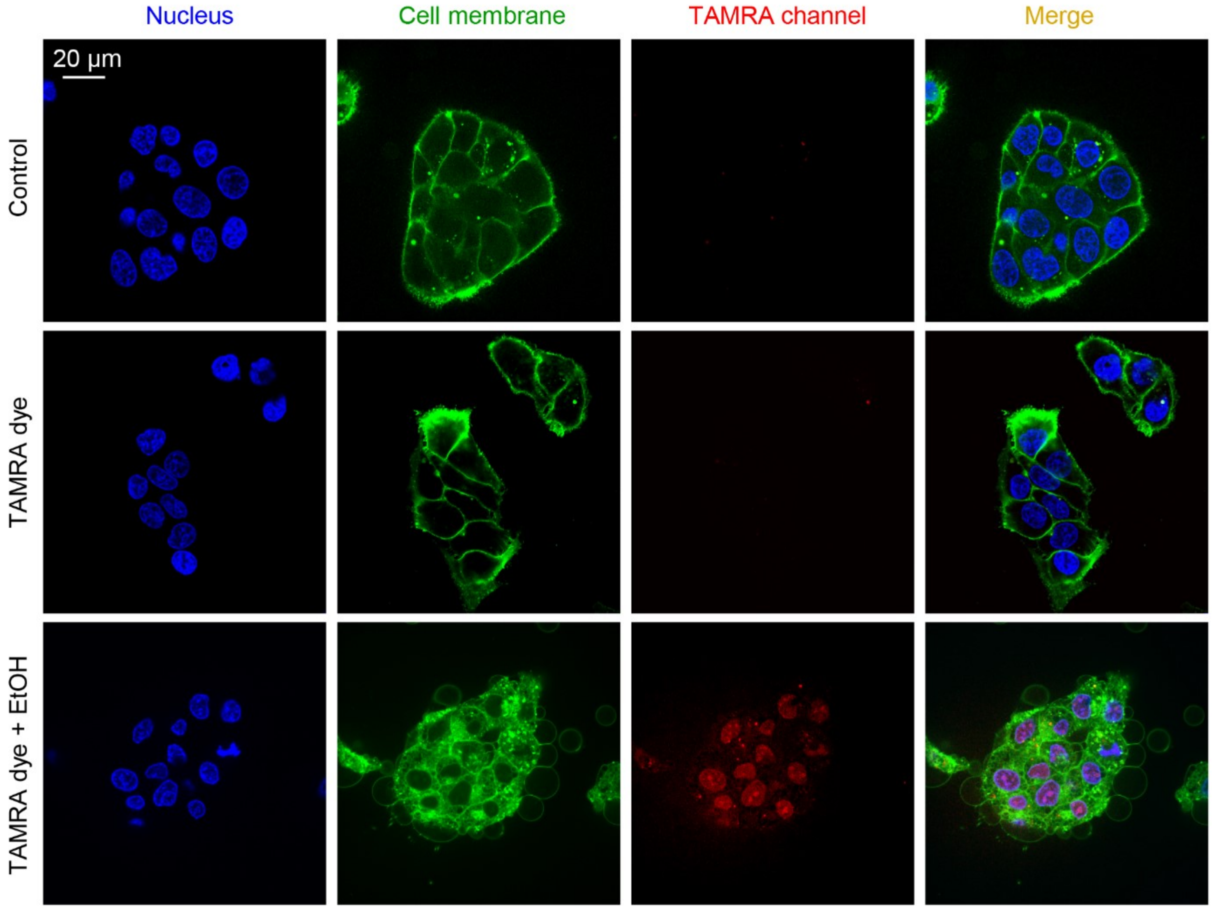

Figure S8. TAMRA dye uptake by HepG2 cells. Cells were incubated with a 50 nM concentration of TAMRA dye for 24 h. After the incubation, plasma membrane was labelled using CellMask Green (Thermo Fisher Scientific) fluorescent probe. Then, cells were imaged using spinning disk confocal microscopy IXplore SpinSR (Olympus, Tokyo, Japan). Hoechst 33342 (blue) dye was

used to counterstain nuclei. Plane images were processed using ImageJ software (NIH). To highlight membrane impermeability of TAMRA, cells were stressed with 20% of ethanol (EtOH).

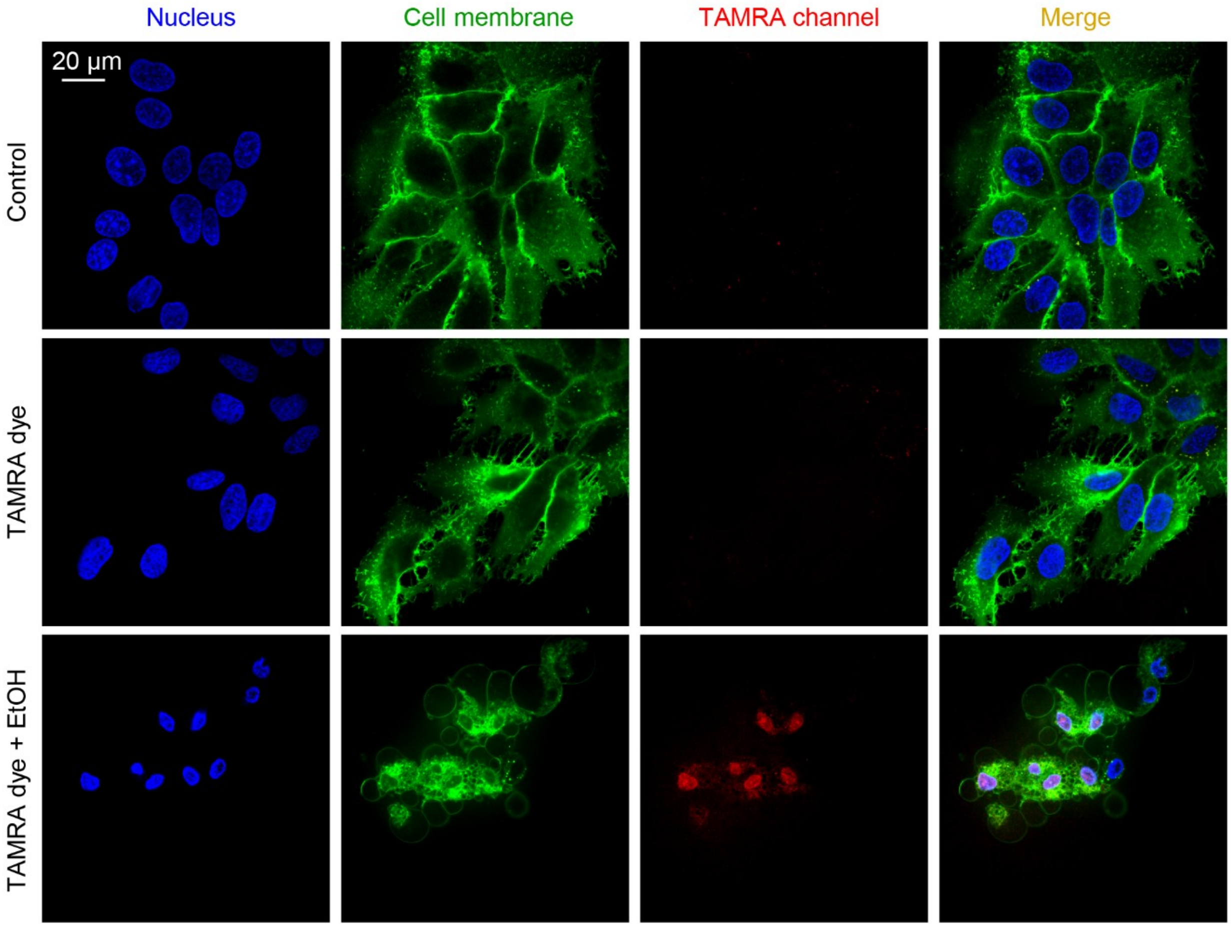

Figure S9. TAMRA dye uptake by Huh7 cells. Cells were incubated with a 50 nM concentration of TAMRA dye for 24 h. After the incubation, plasma membrane was labelled using CellMask Green (Thermo Fisher Scientific) fluorescent probe. Then, cells were imaged using spinning disk confocal microscopy IXplore SpinSR (Olympus, Tokyo, Japan). Hoechst 33342 (blue) dye was used to counterstain nuclei. Plane images were processed using ImageJ software (NIH). To highlight membrane impermeability of TAMRA, cells were stressed with 20% of ethanol (EtOH).

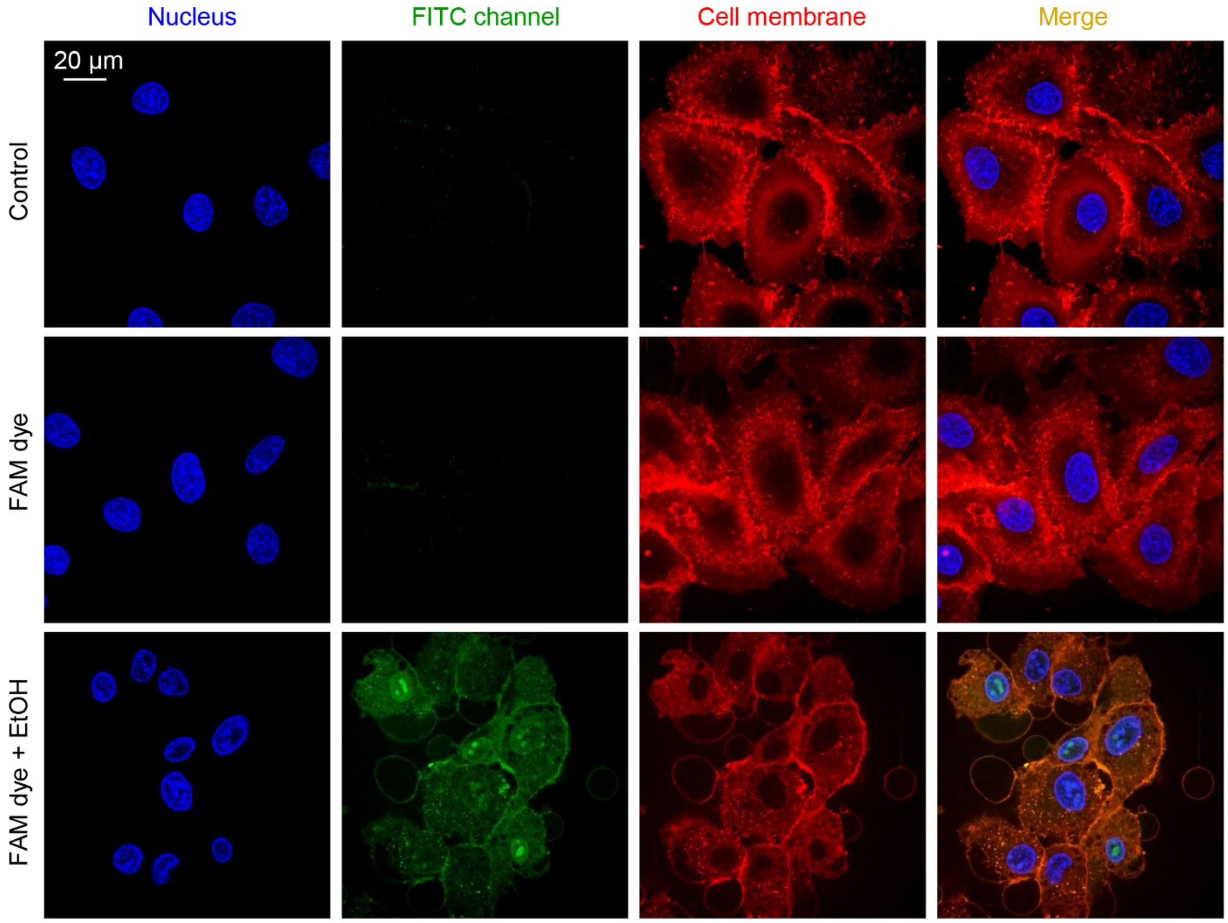

Figure S10. FAM dye uptake by Alexander cells. Cells were incubated with a 50 nM concentration of FAM dye for 24 h. After the incubation, plasma membrane was labelled using CellMask Orange (Thermo Fisher Scientific) fluorescent probe. Then, cells were imaged using spinning disk confocal microscopy IXplore SpinSR (Olympus, Tokyo, Japan). Hoechst 33342 (blue) dye was used to counterstain nuclei. Plane images were processed using ImageJ software (NIH). To highlight membrane impermeability of FAM, cells were stressed with 20% of ethanol (EtOH).

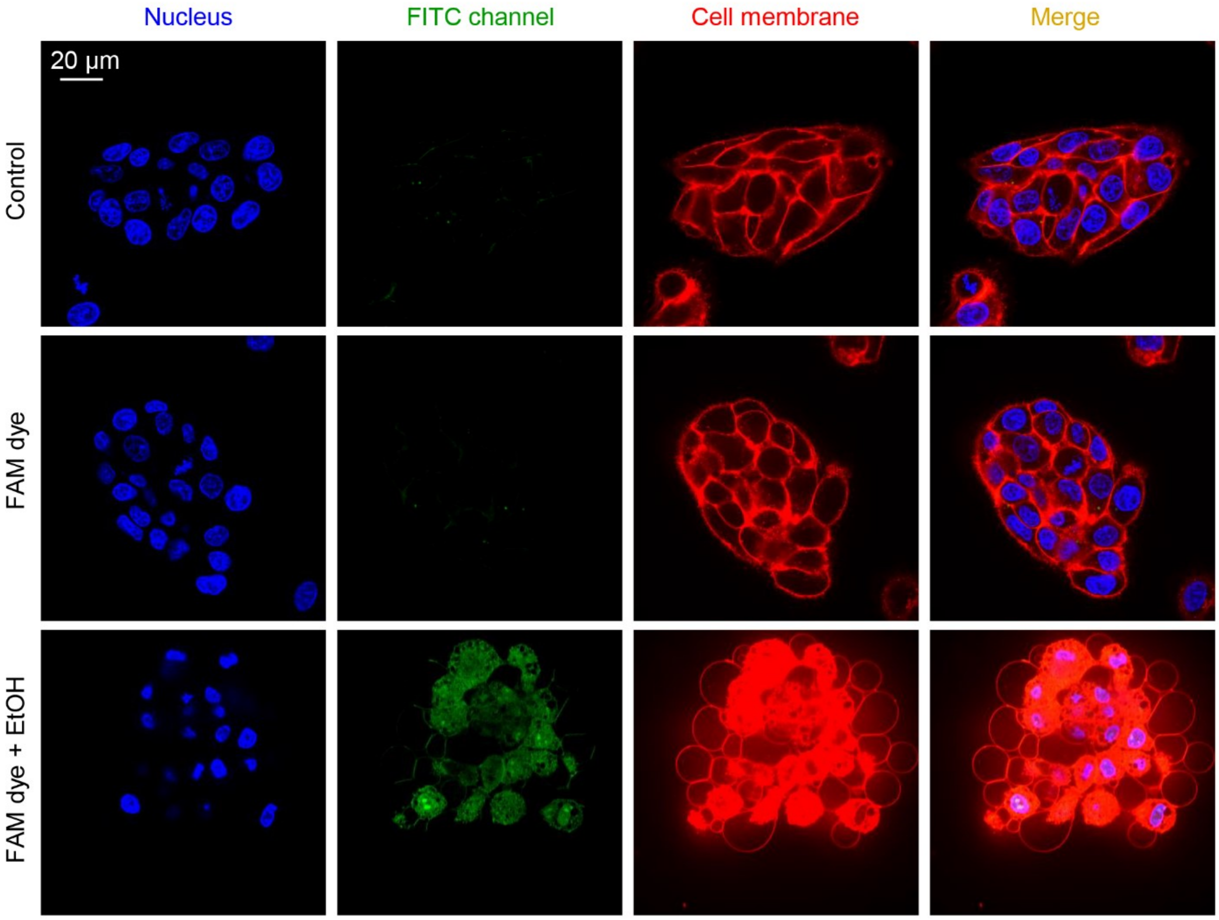

Figure S11. FAM dye uptake by HepG2 cells. Cells were incubated with a 50 nM concentration of FAM dye for 24 h. After the incubation, plasma membrane was labelled using CellMask Orange (Thermo Fisher Scientific) fluorescent probe. Then, cells were imaged using spinning disk confocal microscopy IXplore SpinSR (Olympus, Tokyo, Japan). Hoechst 33342 (blue) dye was used to counterstain nuclei. Plane images were processed using ImageJ software (NIH). To highlight membrane impermeability of FAM, cells were stressed with 20% of ethanol (EtOH).

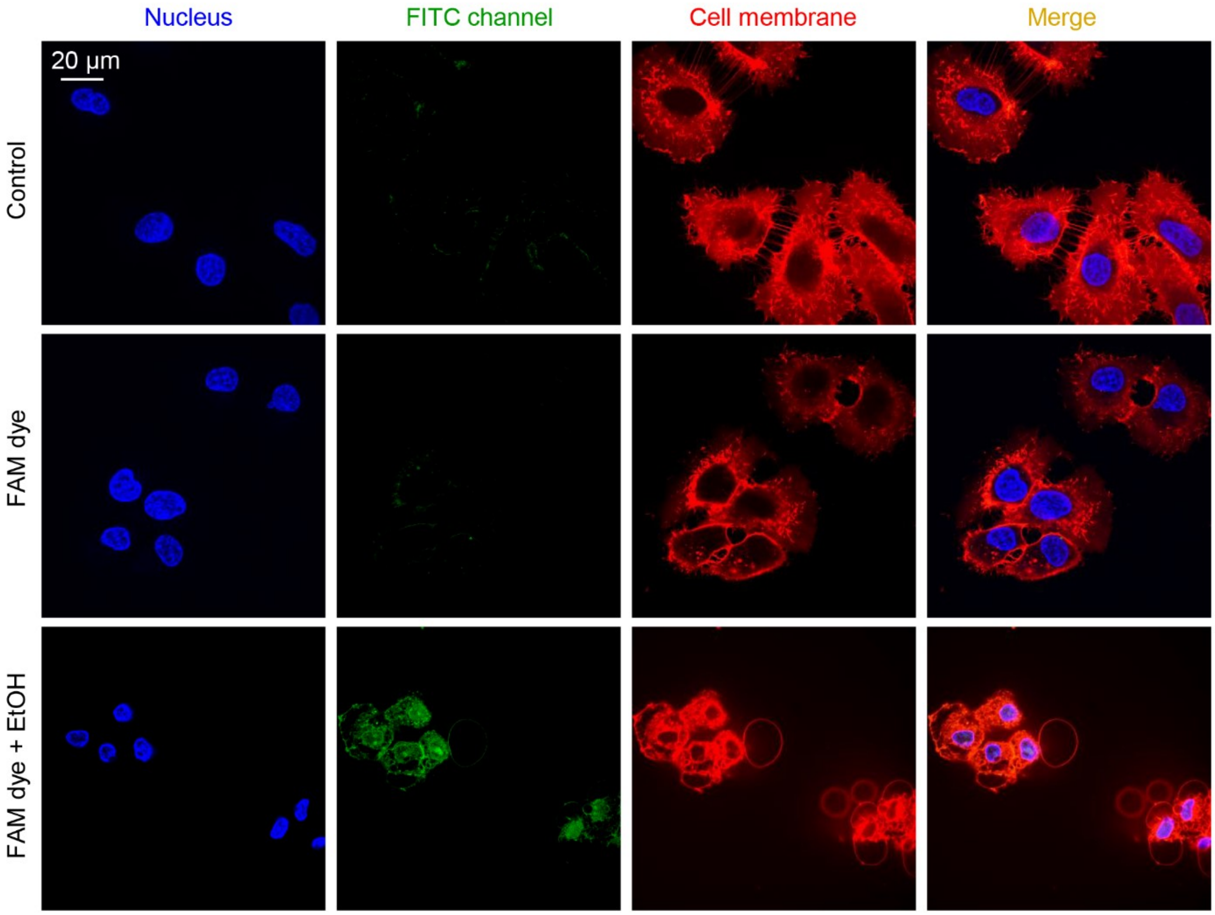

Figure S12. FAM dye uptake by Huh7 cells. Cells were incubated with a 50 nM concentration of FAM dye for 24 h. After the incubation, plasma membrane was labelled using CellMask Orange (Thermo Fisher Scientific) fluorescent probe. Then, cells were imaged using spinning disk confocal microscopy IXplore SpinSR (Olympus, Tokyo, Japan). Hoechst 33342 (blue) dye was used to counterstain nuclei. Plane images were processed using ImageJ software (NIH). To highlight membrane impermeability of FAM, cells were stressed with 20% of ethanol (EtOH).

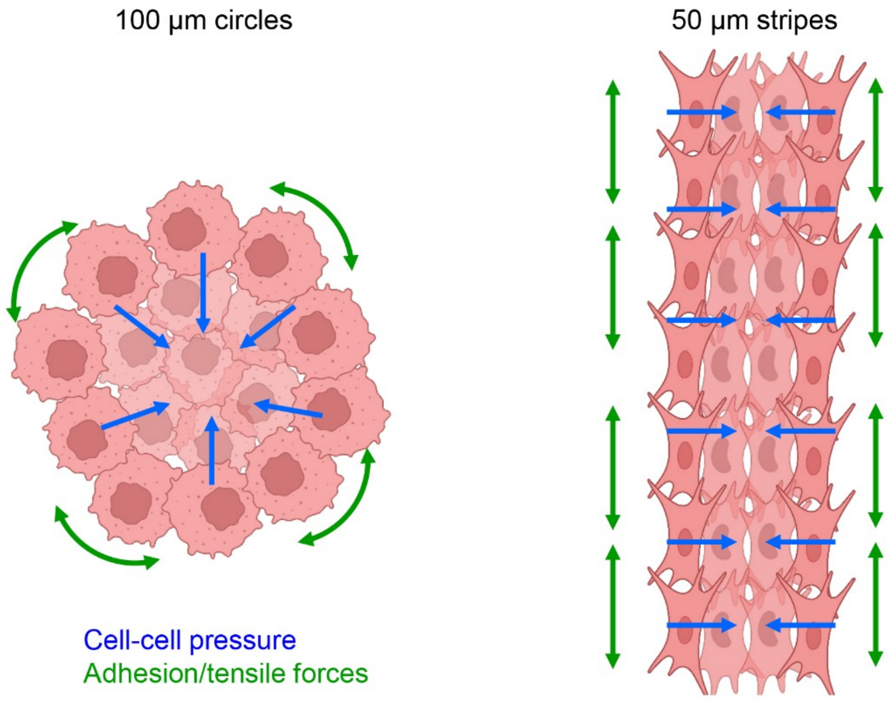

Figure S13. Schematic of how cells within patterned shapes experience competing mechanical cues with varying distribution anisotropy. Geometrical constraint predisposes distribution of mechanical forces according to the patterned geometry. Created with BioRender.com.

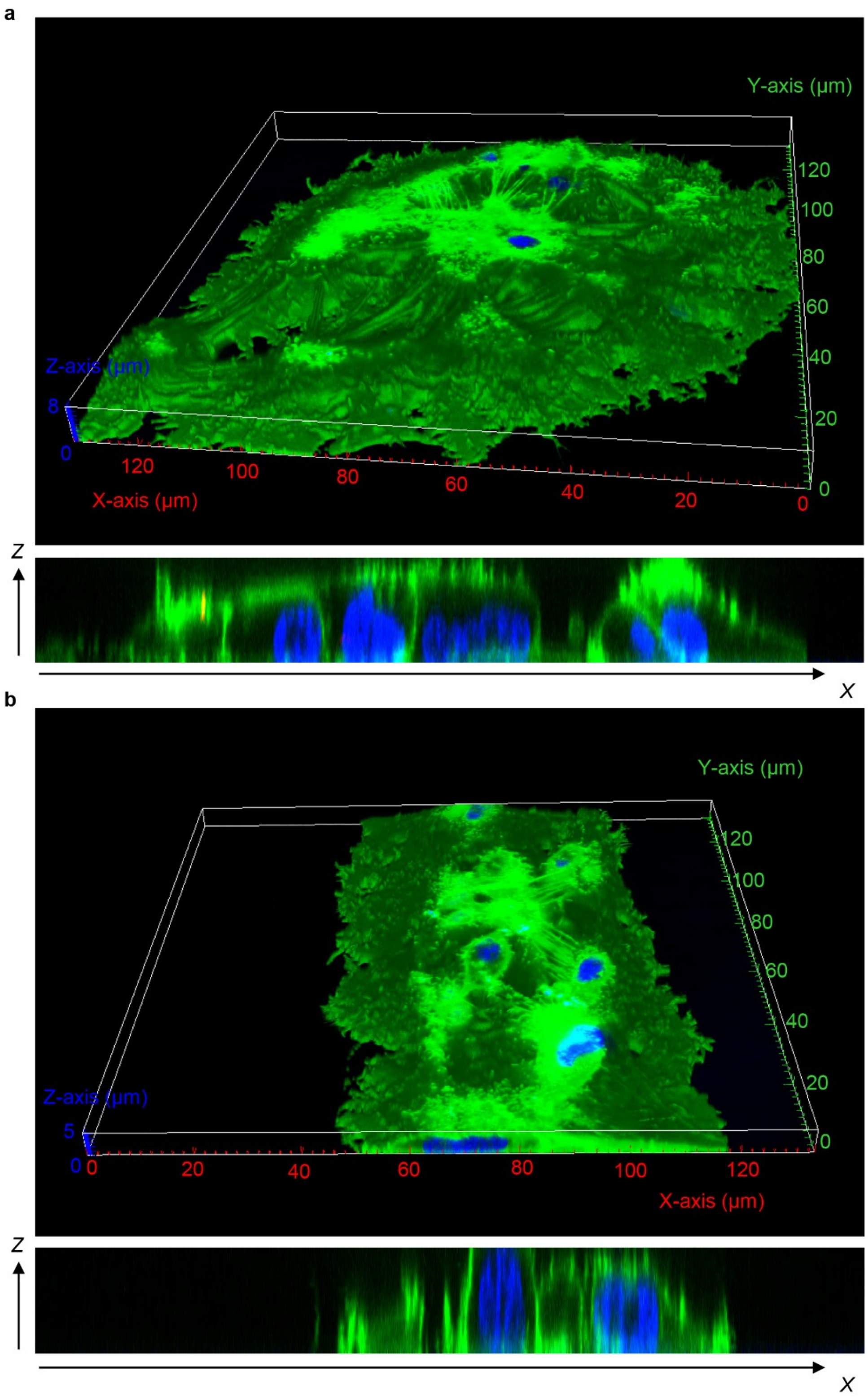

Figure S14. 3D reconstruction of cell surface of cells cultivated on micropatterns (µ-Slide VI 0,4 µ-Pattern^{RGD} Test Patterns 1 (ibidi, Martinsried, Germany, cat. no. 83651). Huh7 cells cultivated

on adhesive micropatterns of various geometry 100 μm circles (a) or 50 μm stripes (b) were treated with 50 nM TAMRA-fluorescently labelled (red fluorescence) 6HB-DNs for 24 h. After the incubation, plasma membrane was labelled using CellMask Green (Thermo Fisher Scientific) fluorescent probe. Then, cells were imaged using spinning disk confocal microscopy IXplore SpinSR (Olympus, Tokyo, Japan). Hoechst 33342 (blue) dye was used to counterstain nuclei. 3D visualization and rendering orthogonal projections were performed using open-source software Icy (https://icy.bioimageanalysis.org).

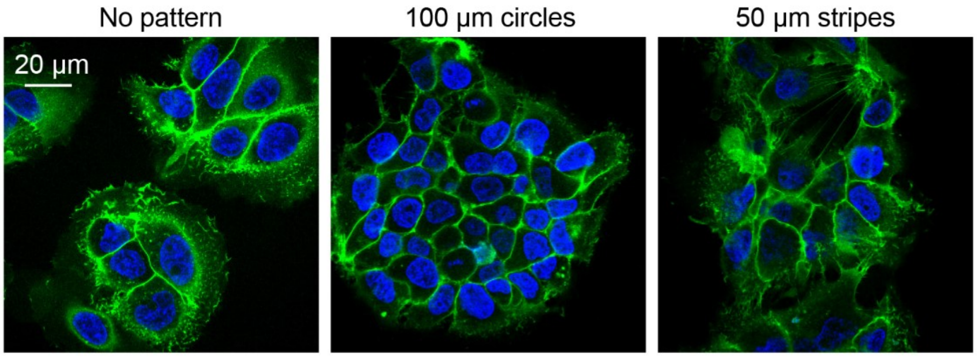

Figure S15. Comparison of cell shape and size between cells grown under standard culture conditions and those subjected to geometric constraints. Huh7 cells were cultivated either under standard culture conditions (6-channel μ-Slides IV 0,4 (Ibidi, Martinsried, Germany, cat. no. 80606) or on adhesive micropatterns of various geometry 100 μm circles or 50 μm stripes. Plasma membrane was labelled using CellMask Green (Thermo Fisher Scientific) fluorescent probe. Then, cells were imaged using spinning disk confocal microscopy IXplore SpinSR (Olympus, Tokyo, Japan). Hoechst 33342 (blue) dye was used to counterstain nuclei. Plane images were processed using ImageJ software (NIH).

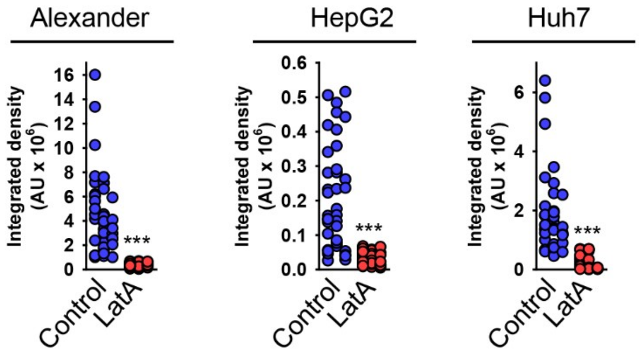

Figure S16. DNs uptake inhibition by latrunculin A. Alexander, HepG2, and Huh7 cell lines were incubated with a 50 nM concentration of fluorescently labelled (green fluorescence) 6HB-FAM-DNs in the presence or absence of latrunculin A for 24 h. After the incubation, plasma membrane was labelled using CellMask Orange (Thermo Fisher Scientific) fluorescent probe. Then, cells were imaged using spinning disk confocal microscopy IXplore SpinSR (Olympus, Tokyo, Japan). Quantitative 6HB-DNs uptake assessment of 35-41 individual cells was performed using the ImageJ macro "Particle_in_Cell-3D" [1]. (***) $P < 0.001$ denote significant differences.

References

1. Blechinger, J., et al., *Uptake kinetics and nanotoxicity of silica nanoparticles are cell type dependent.* Small, 2013. **9**: p. 3970-3980.
